# Quarry mining or nature conservation, emerging conflicts in Serra de Aire e Candeeiros Natural Park (Portugal)

**DOI:** 10.12688/openreseurope.19589.1

**Published:** 2025-11-11

**Authors:** María Guillermina Diaz, Luis Carreira dos Santos

**Affiliations:** 1 Instituto Argentino de Nivologia Glaciologia y Ciencias Ambientales, Mendoza, Mendoza Province, 5500, Argentina; 2 Instituto Politecnico de Tomar Escola Superior de Tecnologia de Tomar, Tomar, Santarém District, 2300-313, Portugal

**Keywords:** Quarry mining, environmental impact, socio-territorial conflicts, PNSAC, natural resources

## Abstract

**Background:**

Recent literature highlights the long-standing presence of environmental conflicts related to mining in Portugal, with cases dating back to the 19th century. While various regions have experienced socio-environmental mobilisations, research has primarily focused on the impact of metalliferous mining on local development. In contrast, studies on the Serras de Aire e Candeeiros Natural Park (PNSAC) primarily address the economic potential of ornamental limestone extraction, often overlooking associated environmental conflicts. Furthermore, despite the park's protected status and its integration within the Natura 2000 Network, few works explore the tensions between quarrying activities and environmental conservation. This reveals a notable gap in the literature regarding non-metalliferous mining and its socio-environmental implications in PNSAC—a gap this study seeks to address.

This study examines the socio-territorial conflicts between quarry mining and nature conservation in Portugal’s Serra de Aire e Candeeiros Natural Park, a protected area facing competing land-use demands. The research aims to address gaps in the understanding of conflicts related to non-metalliferous mining and their socio-environmental impacts.

**Methods:**

Using qualitative content analysis, this study explores semi-structured interviews with key stakeholders. The snowball sampling technique is employed to identify relevant actors and assess their relationships.

**Results:**

The findings reveal tensions between mining companies, park authorities, and local communities, which lead to territorial disputes. The study highlights the challenges of integrating mining and conservation in land-use policies, as well as the lack of clear benefits from local development initiatives. Additionally, it emphasizes the unequal distribution of environmental and social costs associated with extractive activities.

**Conclusion:**

The research highlights the necessity for improved conflict management strategies in protected areas, contributing to broader discussions on sustainable land-use planning. It advocates for policies that balance economic interests with conservation efforts while addressing socio-territorial inequalities.

## Lay summary

**What this study is about:**

This research looks at a problem in Portugal's Serra de Aire e Candeeiros Natural Park, a protected area famous for its beautiful caves and limestone landscapes. For a long time, this park has been a place where two important activities clash: quarry mining (digging up rock like limestone) and nature conservation (protecting the environment).

While mining brings economic benefits, it also changes the landscape and can harm the environment and local livelihoods alike. The park is protected by law, but mining has been happening there for decades, leading to tensions among mining companies, park authorities, and local communities. This study aims to understand these conflicts better, especially regarding limestone quarrying, which hasn't been studied as much as other types of mining in Portugal.

**Why this study is important:**

Understanding these conflicts is crucial because it helps us find better ways to manage protected areas like Serra de Aire e Candeeiros. It highlights the challenge of balancing economic and social needs with environmental protection, especially when different groups have competing interests for the same territory. By identifying these issues, the study hopes to inform policies that support both local communities and the unique natural heritage of the park, thus promoting land use planning practices in protected areas management.

**What the researchers did:**

The research used in-depth interviews with key people involved, such as representatives from mining companies and the Natural Park staff. It also reviewed official documents and used mapping software to understand where mining operations are located within and around the park. This helped to gather detailed information about the conflicts and the different viewpoints of those affected.

**What the researchers found:**

The study found clear tensions between mining companies, park authorities, and local communities. Even though the park has strict conservation rules, mining activities continue to cause environmental damage and socio-economic challenges for local communities.

## Key findings include

***Land Use Conflicts:*** There are clear disagreements over how the land should be used, with mining companies focused on extraction and park authorities on conservation.

***Limited Local Benefits:*** Despite the economic activity, mining doesn't create many local jobs due to advanced machinery, and local communities often don't see significant direct benefits.

***Unequal Burdens:*** The environmental and social costs of mining (like pollution and damage to private property, mainly from transport) are often borne by local communities, while the benefits are exported internationally.

***Complex Relationships:*** There's a complicated relationship where mining companies sometimes contribute to road improvements within the park, and the park, in turn, facilitates the expansion of mining areas.

## What this means for the future

This research highlights the urgent need for better ways to manage conflicts in protected areas. It suggests that public policies should aim to balance economic interests with conservation efforts, while also addressing the inequalities faced by local communities.

The findings show that participatory land use planning involving the entire community is essential to protect natural areas such as the Serra de Aire e Candeeiros Natural Park for future generations.

## Introduction

In Portugal, non-metalliferous mining activity is carried out entirely by nationally owned companies, with around 90% of production intended for export, predominantly towards the Asian market. In 2021, Portugal's commercial production of aggregates, cement, lime minerals, ornamental rocks, and industrial minerals totalled 66,379,493 tonnes, generating a revenue of €459,632. (10 ^3€^) (DGEG - Statistics of Geological Resources of the DSEF-RG).

While mining activities significantly contribute to the economy, it is essential to recognize the serious environmental and social impacts of mineral extraction on the affected areas. Especially when the geological distribution of a concentrated mineral represents a substantial number of active or previous exploitations in a given area. Minerals can only be extracted from their natural occurrence locations, often inadequate for other land uses. This situation favours quarry mining and promotes significant impacts on the surrounding areas ^1^. Consequently produces competition for land, which is in turn reinforced by the site selection of an extractive activity where several factors such as resource availability, costs, socio-economic and environmental factors are key to its expansion ^2^.

The confluence of mining, an activity that by its very nature permanently modifies landscapes, with conservation, which seeks to preserve or restore sustainable natural production systems, represents a challenge for environmental and land-use planning policies ^3^.

The current European legal and regulatory framework for environmental protection allows mining activities in natural protected areas. The European Commission states that there is no automatic ban on Non-Energy Extractive (NEE) industry activities within or near the Natura 2000 network. Activities related to mining must comply with Article 6 of the Habitats Directive. They are subject to an appropriate assessment to ensure that they do not harm the integrity of Natura 2000 sites. Furthermore, each mining project must be reviewed individually, taking into account its specific conditions and potential impacts.

The Natural Park (NP) of Serras de Aire e Candeeiros presents a unique situation within the Natura 2000 Network, further classified as a Natural Park. Its primary goal is to protect the natural values of the Extremadura Calcareous Massif, though it also permits the expansion of mining activities by allowing the rehabilitation of abandoned quarry sites as part of the restoration purposes. In this sense, 4 Machado *et al.* (2014) identifies as a *difficult coexistence* generated between the natural heritage and the aggregate extractive industry that has been developed in the park before its classification.

Forty-five years since the park's establishment, a thin line continues to separate nature conservation from the growing mining activities. The lack of clear outcomes from local development initiatives is evident, as the ageing population and demographic decline become increasingly noticeable in a region shaped by the economic interests of globalization, open markets, and the depletion of natural resources. While the Sustainable Development Goals (SDGs) offer a new perspective on sustainability for the 21st century, deeply rooted traditions and contradictory approaches to sustainable development may pose significant barriers to achieving these important objectives.

Likewise, the clear contradictions that can be interpreted in the Management Plan for the Serra de Aires e Candeeiros Natural Park (POPNSAC) allow us to frame the clash of interests in the area under study, which reveals the environmental conflict generated by the uses that the different actors make of the territory. From this point of view, we define socio-territorial conflicts as situations in which there is a clash of interests between two or more groups of actors over the use and appropriation of natural resources. At this point, we emphasize that socio-territorial conflicts are eminently social and strategic, i.e. they are situations that are triggered over a given time so that temporality is a feature that characterizes them, as well as the measures that social actors adopt as the conflict is unleashed. From this definition, the focus is on the unequal spatial distribution of the negative social and environmental costs and impacts that can be caused by the development of extractive activities by one group of actors over others ^5– 9^.

In this regard, how is the territory restructured by the development of extractive activity in a classified protected area? What influence does territorial configuration have on the emergence of environmental conflicts? These questions serve as a guide for this study, which aims to identify the presence of socio-territorial conflicts in a highly protected area.

## Literature review

Recent academic literature indicates that environmental conflicts related to mining activities in central Portugal can be traced back to the 19th century. As mentioned by 10 the cases of Braçal (1836–1959) and Talhadas (1889–1931), with the exploitation of Copper, Silver, and Lead, other Portuguese regions with lesser mediatic impact were also identified. The same authors unveil mining conflicts in places such as the Algarve: Tavira (Santa Catarina da Fonte do Bispo), Monchique (Carapitotas, Corte Grande); North: Barcelos (Barqueiros, Vila Seca and Milhazes); Center: Coimbra (Cantanhede, Soure, and Mira), Figueira da Foz (Ferreira-a-Nova and Bom Sucesso), Leiria (Pombal), Viseu (18 Municipalities), Sever do Vouga (Braçal mining complex); Águeda (Talhadas mines); Alentejo: Évora (Boa-fé, Serra de Monfurado), Portalegre (Nisa). Using a methodology that combines documentary literature and social analysis, the authors report significant social mobilization and protests that have influenced current legislation ^11^, and analyse the socio-environmental conflicts occurring in Portugal between 1975 and 2015. They examine various factors, including the level of mobilization, media attention, organization, impact, and ideological references that influenced these conflicts during this period.

At the local level, Serra de Aires e Candeeiros has been the subject of several studies on mining, yet observed little to no connection to environmental conflict. Most studies focus on the relationship between this activity and economic development. In this sense, 12 most studies carried out a detailed analysis of the geological conditions of the study area, highlighting its potential for the exploitation of ornamental limestone, claiming it would bring important benefits to regional development.

It is also important to mention that the land-use planning policy for the Serras de Aire e Candeerios Natural Park allows the expansion and exploitation of already functioning companies by granting new licenses to individuals who adopt decommissioned quarry restoration practices. With that in mind, some authors ^12, 13^ proposed a methodology to define and delimit the associated geological suitability areas, aiming to reduce environmental conflicts in the Serras de Aire e Candeerios Natural Park by integrating parks’ objectives.

Ferreira Carvalho *et al.* ^13^ also identified five nuclei, Moleanos, Salgueiras, Cabeca, Veada, Pé da Predeira and Codacal, where ornamental rocks quarrying is carried out in the Natura 2000 Network area within the NP. Concomitantly, the authors highlight the existing problems linked to the compatibility of mining activity with natural environment conservation, as do the actors involved in the process, whom they define as those responsible for the territorial management and knowledge, the public sector, and the private sector (mining companies and their associated entities).

Other studies also reveal the connection between mining activities in the NP and the development opportunities this industry can bring to local communities. By doing so, 14 using a geological approach, carried out a study to unravel the questions linked to the possibility of developing extractive activity of the mineral masses compatible with local development, that is, to think about the possibility of added value that quarries could bring to territories in which they are located. The author seeks to contribute to the identification of the challenges that mining operations represent for local territorial planning and development.

All things considered, the literature reviewed allowed us to demonstrate the existence of environmental problems and conflicts caused by mining activities occurring both on a national and local scale, where competition arises between mining operations and communities residing in protected areas, such as the ones in PNSAC. However, the cited literature focuses mostly on local development linked to cases of conflict around metalliferous mining activity as opposed to the low relevance given to quarry mining.

Moreover, the above-cited works bring to evidence the literature gap linked with non-metalliferous mining activity and environmental conflicts, particularly for the PNSAC area, hence why this paper aims to contribute to contribute towards filling this gap in literature.

## Study area

Portugal is located on the Iberian Peninsula, covering an area of 92,061 km², which includes a continental part measuring 88,944 km². The Extremadura Calcareous Massif (ECM) spans an area of 900 km² in central Portugal, approximately 100 km from Lisbon. This massif consists of a thick sequence of Mesozoic carbonate rocks that are structurally elevated. The lithostratigraphy of the area is well understood, with primary ornamental rock varieties dating back to the Middle Jurassic period, specifically the Batonian and Calovian ages. The ECM has five main locations where extractive activities occur: the Pé de Pedreira, Moleanos, Codacal, Fátima, and Alvados areas.

The highlands of this limestone massif are classified as a natural protected area comprising the D’Aire and Candeeiros mountains, which hold most of the extractive industry.

**Figure 1.**
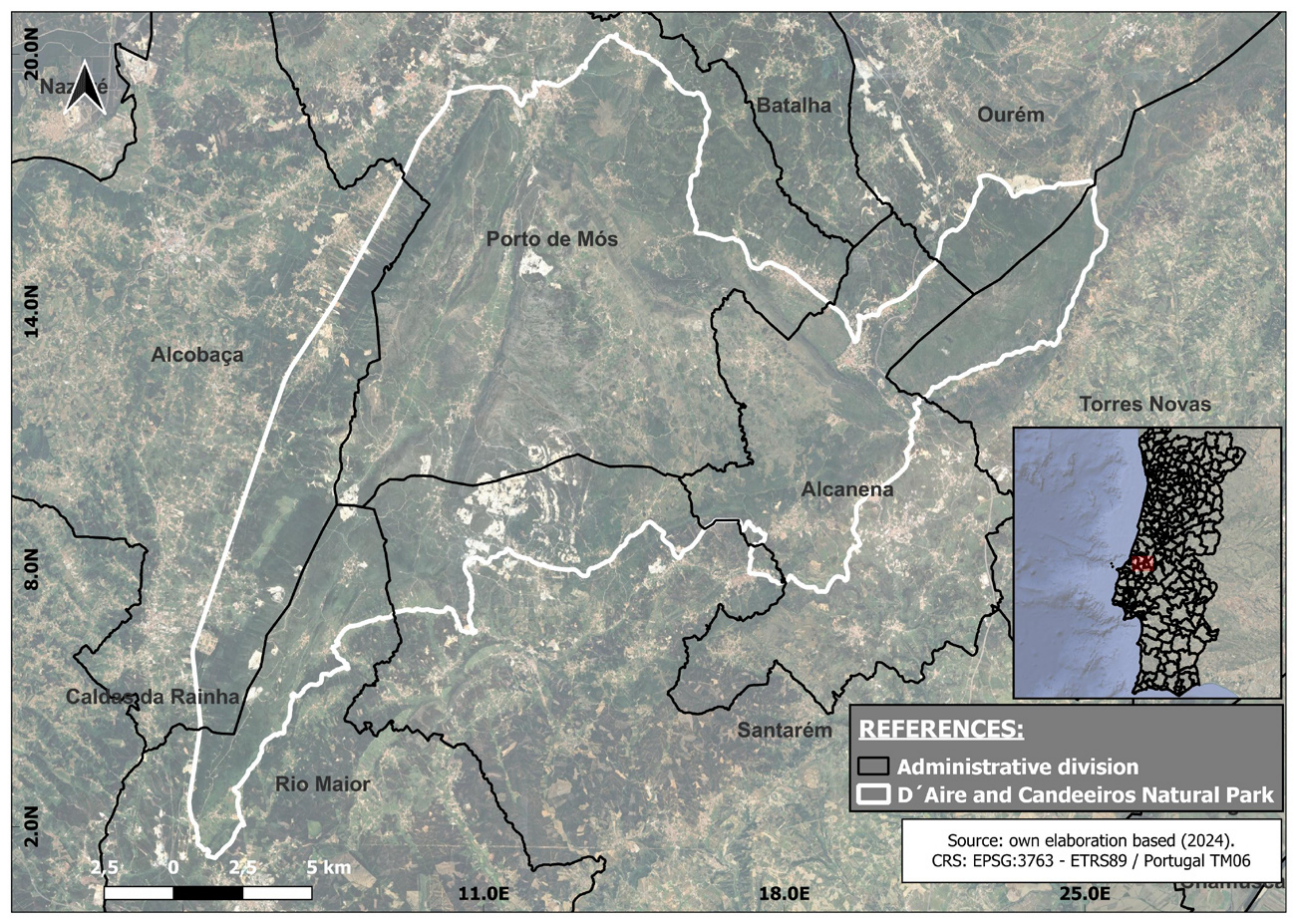
Geographical location map. The black lines show the administrative division of the region under study, and the white lines delimit the area comprising the Serra D'Aires e Candeerios Natural Park.

Administratively part of the Santarém and Leiria Districts and covering seven municipalities, in the district of Santarém, the municipalities of Alcanena, Rio Maior, Santarém, Torres Novas, and Ourém, in the district of Leiria, are the municipalities of Alcobaça and Porto de Mós ( Figure 1). The NP of Serras D’Aire e Candeeiros is one of the 13 Natural Parks of Portugal. The NP of Serras de Aire e Candeeiros was declared in 1979 by decree-law (N°18/79) with the main objective of safeguarding the natural and heritage values of the ECM, also holding classified areas by the Natura 2000 Network.

Located in the centre-west of Portugal and covering an area of approximately 38.900 ha the D´Aire and Candeeiros mountain are the most important reservoir of limestone formations in Portugal, their karstic morphology, the nature of the vegetation cover, the network of underground caves and watercourses, the endemic fauna and flora are the main aspects that contributed towards the classification of this area as a NP. Nonetheless, the abundant limestone natural resources undergo an intense pressure from the activity in the field of mineral resource extraction.

In terms of orographic features, four zones can be distinguished: two mountain ranges, the D'Aire (678 m) and the Candeeiros (615 m), and two plateaus, the Santo António and the São Mamede ^15^. There are numerous underground karst formations and some notable features, such as the *poldje* of Aire-Minde, Mira, and Mendiga dolines. Lastly, the park also features several landforms that result from the dissolution of rock due to water infiltration through diaclases.

Climatically, 16 states that Portugal's climate is mainly temperate Mediterranean. The peculiar characteristics of the Park's microclimate are: first, the thermometric characteristics, the average annual temperature registers values between 13°c and 15°c. The high summer temperatures are softened or tempered by the proximity of the Atlantic Ocean, resulting in July being the warmest month, with an average temperature varying between 18° and 24°C. The significant winter rainfall deserves attention, with annual averages ranging from 800 to 1,200 mm.

Regarding the area's biogeography, 16 notes that the vegetation defining the PNSAC stems from the Mediterranean bioclimatic influences on the region, predominantly rocky areas with lush vegetation on limestone summits, slopes with tree cover, scrubland, undergrowth (garrigue), and orchards found in depressions.

The aforementioned natural attributes are conducive to developing agricultural and livestock activities located in the main fertile urbanized depressions.

In demographic terms, according to data from the Portuguese National Institute of Statistics ^1^, by 2021 the park´s municipalities will have approximately 20,000 inhabitants, mainly in the valleys surrounding the Sierras de Aire and Candeeiros and below the plain of San Antonio. This accounts for the scarce presence of a stable population living within the Park.

## Method

The nature of this research compelled a specific methodological design consisting of three stages:

1- The first stage involved the delimitation of the study area and the definition of the subject participants.
2- The second stage, considered the search for extractive companies operating in the region and data collection (semi-structured interviews).
3- Finally, the third stage consisted of data sorting and analysis.

The initial approaches to the study area were made through the use of a technical literature review, reviewing different documents: the Territorial Management Plan of the Serra de Aire e Candeeiros Natural Park, and searching for official statistical data published by the Portuguese General Directorate of Energy and Geology (DGEG).

Subsequently, geo-referenced information was used to map the total area comprising the PNSAC, the mining area inside the park, and its adjacent boundaries. The software QGIS 3.26.1 Buenos Aires version was used for this purpose. This resulted in the identification of the mining areas within the park, determining that the area to be covered during the fieldwork is included within the municipalities of Alcanena, Alcobaça, Ourém, Porto de Mós, Rio Maior, Santarém, and Torres Novas ( Figure 1).

Once the area was established, contacts were made with extractive companies, considering those willing to provide information on possible socio-territorial conflicts and environmental issues in the geographical study area. Based on the delimitation of the study area, the mining companies operating within the PNSAC were selected.

It should be noted that when working with a population for which there is insufficient information to define a representative sample, the snowball technique was used to estimate population size and understand central aspects such as the types of links they have with each other ^17^.

Extractive activity, as a business, is bound by hermeticity and competition in this economic sector. As stated in the existing literature, the population was defined as hard-to-reach populations ^18– 21^.

The term hard-to-reach population refers particularly to populations that do not wish to be contacted or identified based on specific criteria, as well as groups that are considered difficult to reach because of their geographical location. 17, 21 define the groups of people who are sometimes considered difficult to reach or hidden, as those subjects who have no interest in being found or contacted.

The authors state that “In these circumstances, it can be very difficult or expensive to recruit study subjects” recommending the use of snowball sampling method as a reliable referrals-based method, indicating that “initial known subjects to recruit new additional subjects.” ^21^.

Accordingly, 22 argue that a chain-referral sampling method relies on referrals from initial subjects to generate additional subjects.

**Figure 2.**
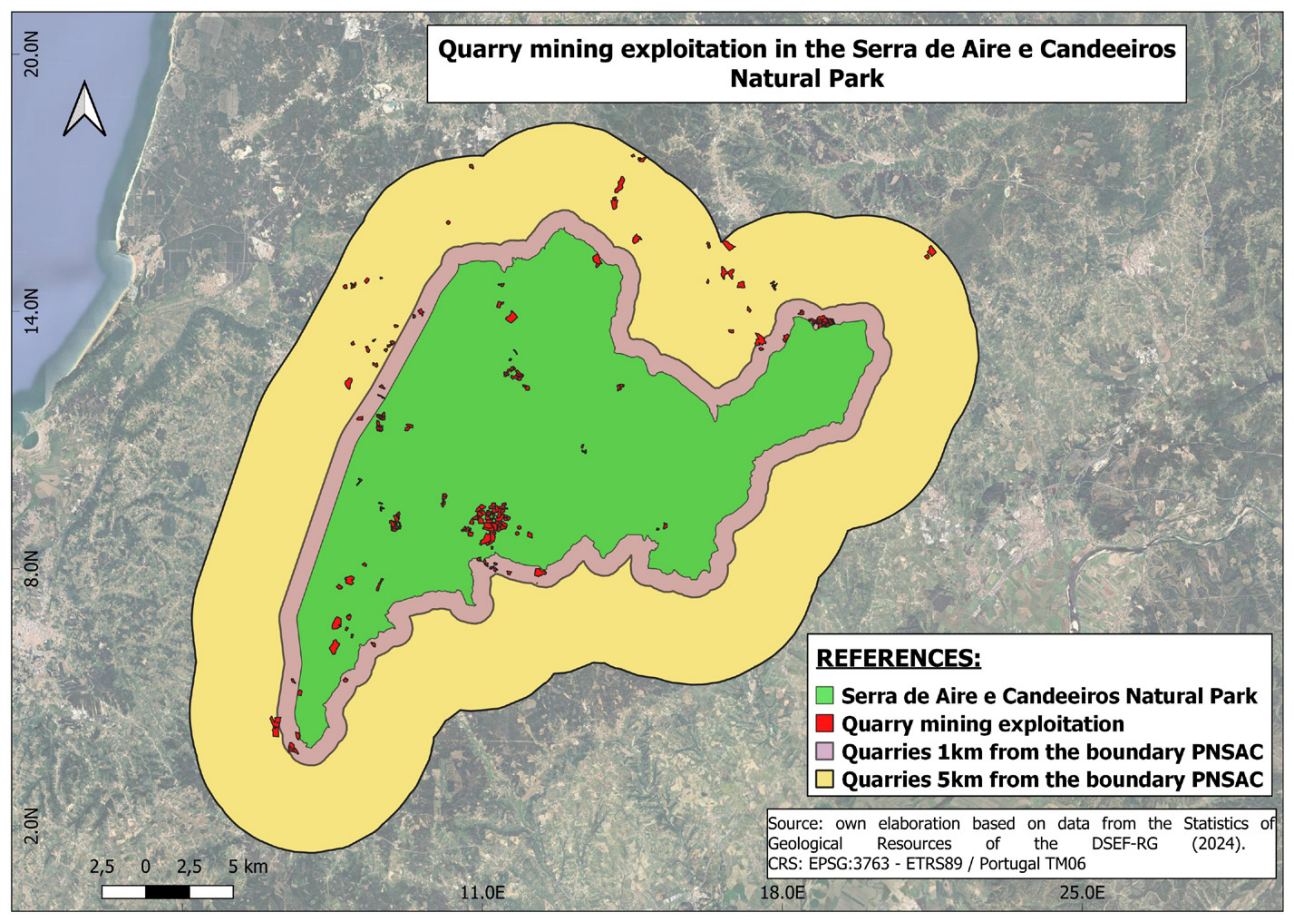
Location of mining operations. The area covered by the Serra de Aires e Candeeiros natural park is shown in green. The red colour shows all the mining operations that operate within the park and its borders. The purple colour shows the distance between the boundary of the park and the mining operations within a distance of 1 km. The yellow colour represents the distance between the park boundary and the mining operations within a distance of 5 km.

The sampling decision was made on the basis of three criteria, the first being that the enterprises must operate within the PNSAC, i.e. the selected mining company must be located within the park, or a 1 km and 5 km radius of the park ( Figure 2); the second criterion is linked to the size of the quarry, between 1 and 15 ha, with 1 ha being defined as small and 15 ha or more as large quarry. The third criterion is the distance to a population centre of up to 5 km ( Figure 2).

Quarry mining data was resampled from the DGEG Portuguese National database using QGIS to select mining companies within the set boundaries. Resulting in 195 quarries being selected, considering both 1km and 5km buffers. Where 39 quarries are located within the 1km buffer, 38 are located between the 1km and the 5km buffer, and 118 quarries are located inside the NP.

**Figure 3.**
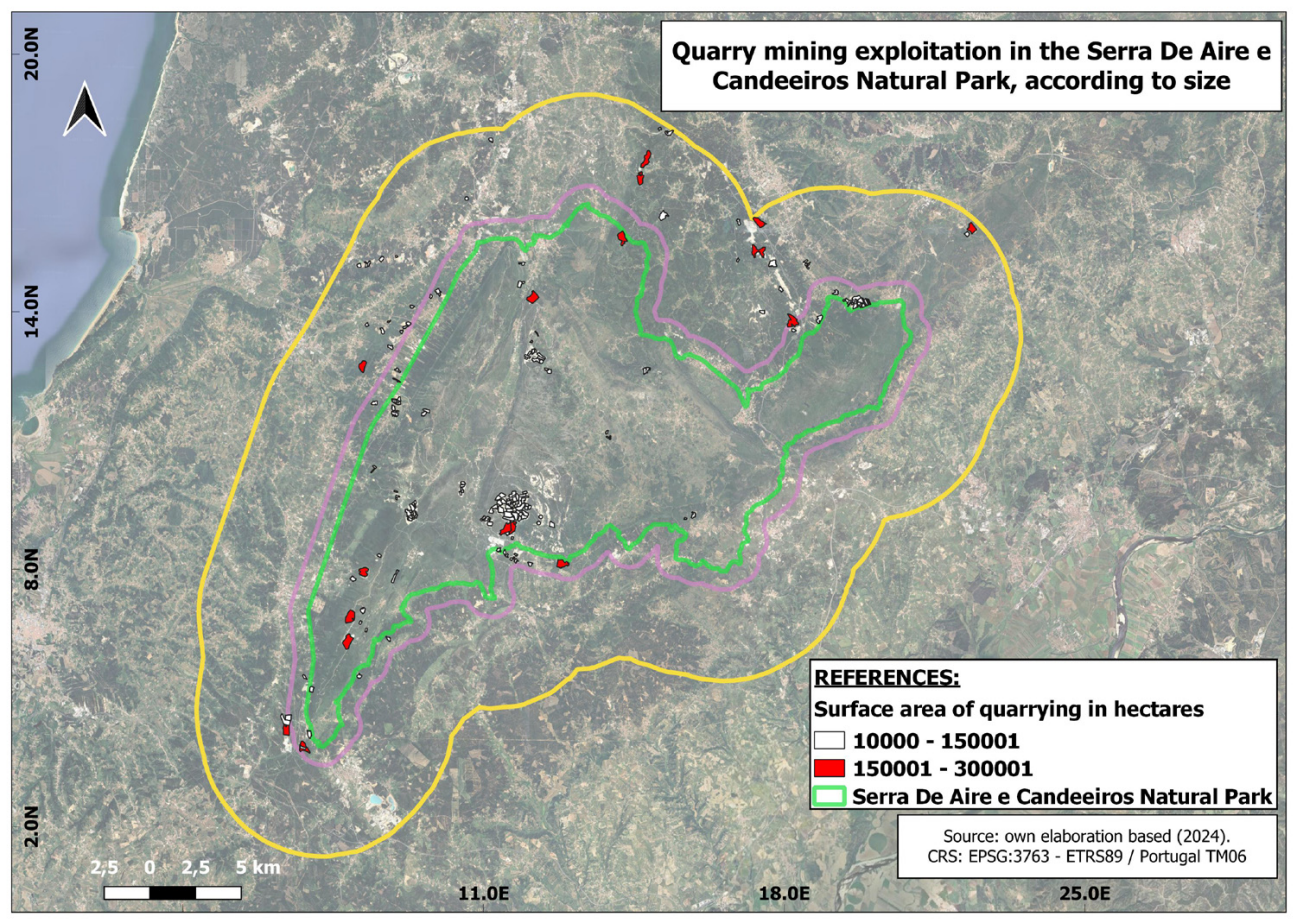
Location of mining sites according to their size. In white colour are represented the mining exploitations that have a surface size between 1 and 15 hectares. In red colour are represented the mines with a surface between 15 and 30 hectares. The green perimeter represents the boundary of the natural park.

In terms of the size of the selected companies, 18 large quarries and 117 small ones were identified. ( Figure 3)

Despite the use of systematic sampling, the snowball technique was used to select the companies to be interviewed. This technique allowed us to identify only 4 quarries as representative samples for the study. Of the five interviews, four were conducted with mining companies within the park and one with Natural Park staff.

In this way, 5 semi-structured interviews were carried out, which aimed to gather information on the activity developed by the quarries, specifically their links with other actors in the area where they operate, without neglecting to mention those issues that brought us closer to the identification of socio-territorial conflicts and the environmental impacts generated by the activity.

Ethical clearance was granted through the Highlands.3 project under European Union Horizon 2020 regulations, ensuring compliance with international research standards. All respondents received a written and oral description of the project in the local language and verbal/recorded informed consent forms. We emphasised to all participants our commitment to the principles of confidentiality and respect for cultural and institutional sensitivities.

To complete the data collection, semi-structured interviews were designed and conducted with the 4 selected mining companies and, in parallel, direct field observation was carried out.

The study area was visited for interviews, and visual data, namely photographs and videos, were collected, generating a valuable photographic and film archive.

## Results and analysis

Based on the interview questions, a list of 9 codes was generated ( Table 1) with their own definitions, allowing the methodological variables to be analyzed to be specified.

**Table 1.** Code definitions. Own elaboration, 2023.

Code	Definition
**Conflict**	Confrontation of interests between different actors in the use of natural resources.
**Environmental Impact**	Water contamination, landscape deterioration, land clearing, pollution.
**Local community**	Liaison with the neighbours of the localities surrounding the quarrying area.
**Production process**	Refers to the form of material extraction.
**Infrastructure**	Construction or maintenance of roads.
**Type of material**	Rocks extracted in the production process
**Market**	To where the production is marketed, i.e. to the international or national market.
**Natural Park**	Existence of links between extractive companies and the park.
**Environmental recovery**	Set of actions carried out by extractive companies to remediate or restore the natural conditions of an environmentally degraded site.

The coding system was created inductively, through the analysis of interview content. This system contains a total of 9 codes ( Table 2). The data analysis was carried out in MaxQDA, through this software the codes in the interviews were identified in order to analyze the qualitative data.

**Table 2.** Coding system resulting from interviews.

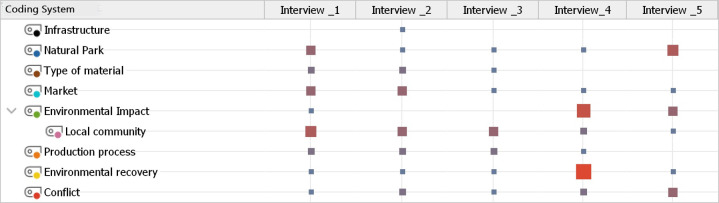

Table 2 shows the weight of each code analyzed in the different interviews, and then the *word count function* and the *sum* were used to analyze the preponderance of the codes in each document.

The utilisation of the devised coding system enables the observation of the relative weight of each code in the interviews. The number of instances of the codes in the documents was considered to ascertain this relative weight. Of the nine codes identified, five have a higher weight, which is represented in the table with a larger size and in dark red: local community, environmental remediation, environmental impact, conflict, and type of material. Conversely, the smaller quadrants indicate a lower relative weight linked to a low presence of the codes in the interviews.

It should be noted that upon the quantification of all summed analyzed variables, the number of variable occurrences in the interviews was calculated for each variable and code. This resulted in a discrepancy between the documents, as some presented a greater number of variables identified in the accounts of the interviewed actors. Additionally, a discrepancy was observed in the coding system itself, where the resulting values allowed for the observation of differences in their frequency in the interviews.

**Figure 4.**
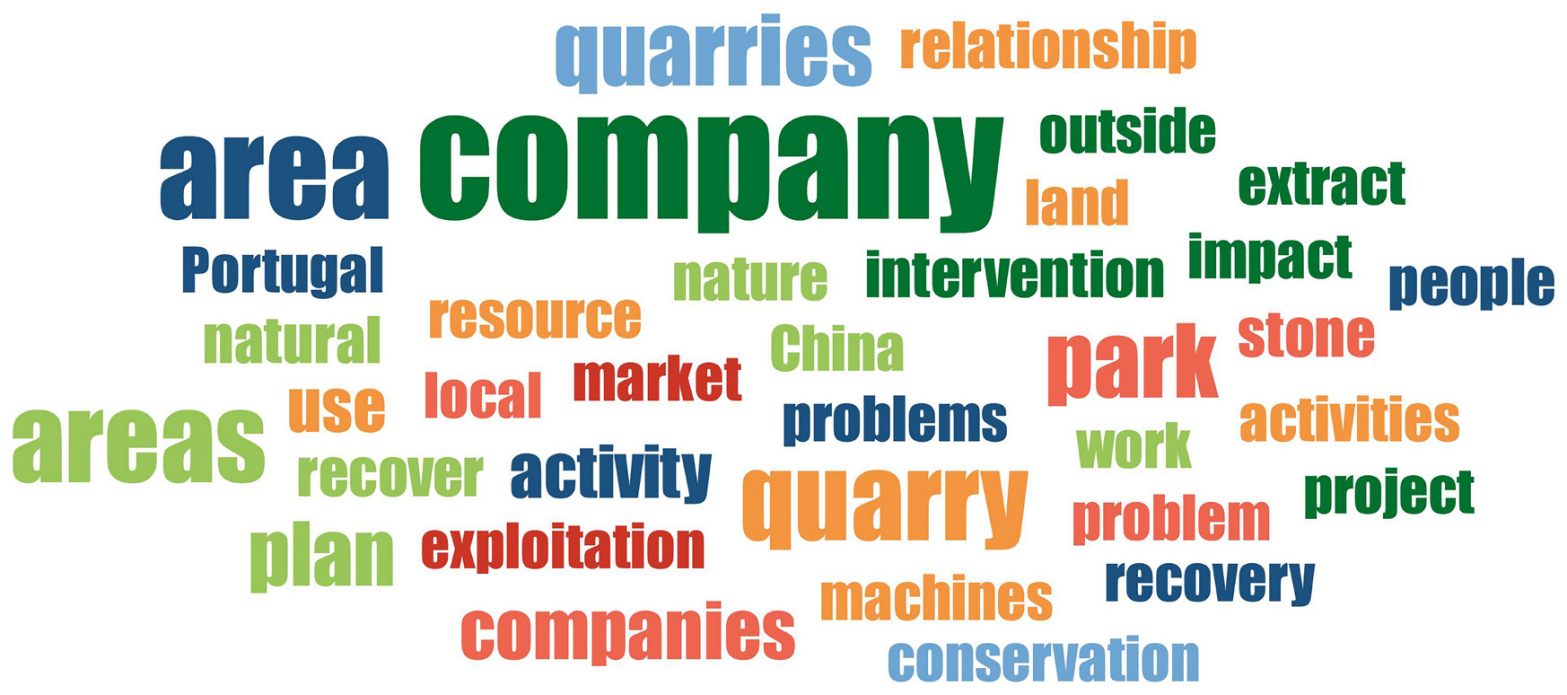
Word cloud created from the thirty-five most representative words obtained from the interviews. The colours are random.

In the context of word frequency analysis, the objective of this task is to ascertain the terms that are most frequently cited by the interviewed subjects, with a view to identify those that are of the greatest pertinence. For the purpose of word frequency count, the fifty most prevalent words were designated as the selection criteria. The list was subjected to three iterations of purification, during which the so-called 'empty words' or words devoid of content were discarded. The following word cloud ( Figure 4) illustrates the thirty-five most representative words (with the highest percentage load).

The word cloud serves to illustrate the presence of companies operating within and outside the PNSAC, and the nature of their activities. Concerning the nature of these activities, quarrying is recognized as an extractive industry, carried out with the use of state-of-the-art machinery and technology to exploit resources, which is associated with the absence of employment for the local communities living in the PNSAC. The word cloud provides a clear illustration of the market(s) to which the production is destined, namely the international and national markets, with exports destined for China and the remainder distributed within Portugal.

Despite the fact that the companies undertake remediation initiatives in areas of exploitation through reforestation or water reuse projects, as evidenced by one of the companies, the word cloud underscores the environmental implications that this type of activity can engender in a protected area through the intervention that is made on its territory. This is further compounded by the challenges that the development of mining engenders in terms of land use among the various stakeholders residing there, giving rise to socio-territorial conflicts.

## Discussions

The analysis indicates that the conflicts are rooted in a specific form of land use, namely open-pit quarrying, which primarily targets ornamental rocks and limestone through the use of the cutting method, using water in the extraction process, with a focus on meeting the demands of an international market. There is a limited presence of community engagement in the surrounding areas, and the nature of the activity is such that it employs a reduced number of individuals, relying predominantly on mechanized solutions for the execution of the production process, limiting employability.

The relationship with the natural park is focused on granting exploitation permits, either to initiate the activity or to reclaim abandoned quarries, to expand the exploitation area by mining companies.

In terms of market analysis, it was observed that companies export 90% of their production, leaving a limited margin for sales and distribution within the Portuguese national market. The Asian market is the primary purchaser of marble blocks extracted by the companies, with the residual material from the production process targeting the national market.

**Figure 5.**
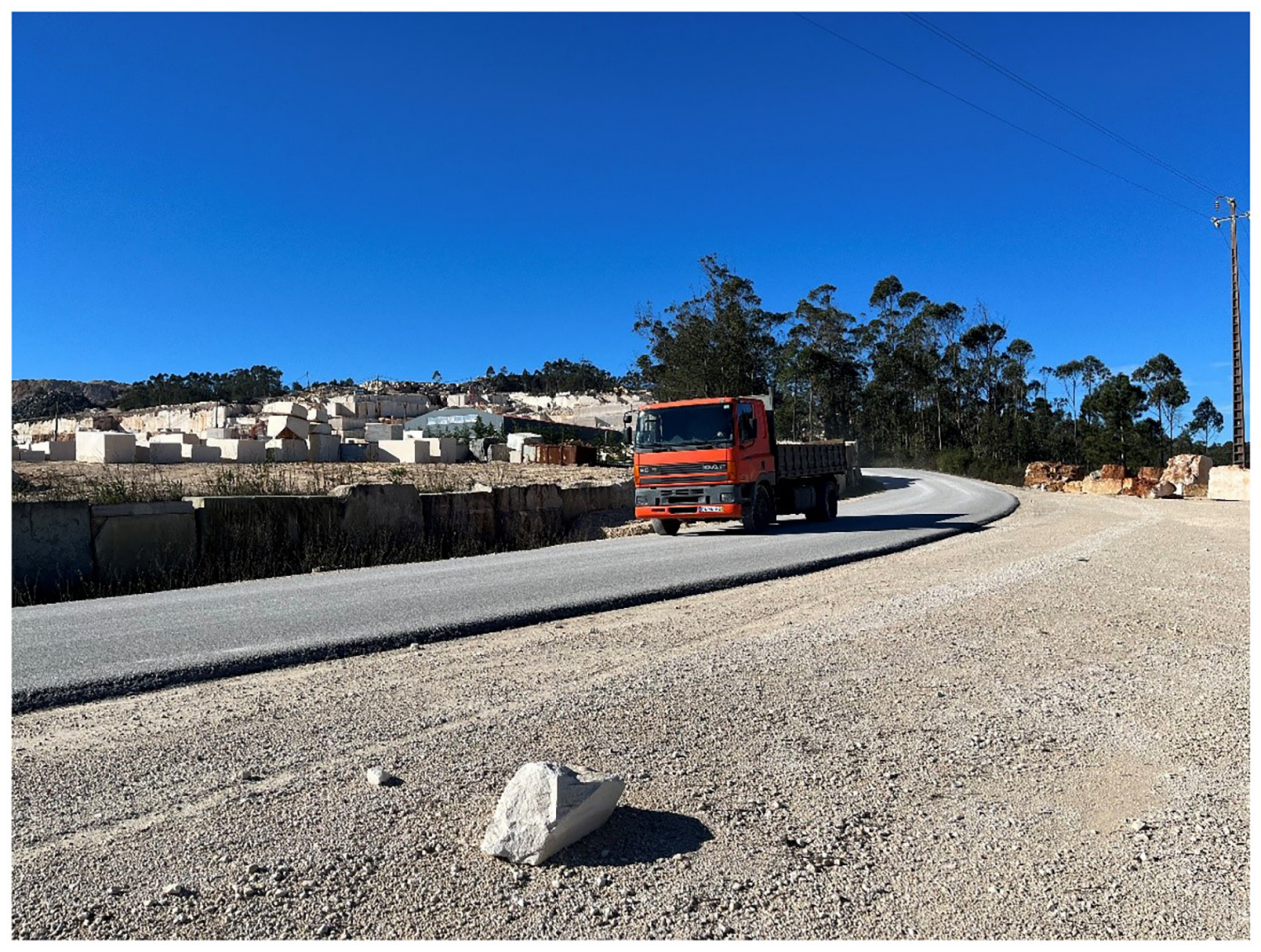
Photograph taken during fieldwork on one of the visits to the mining sites. The vehicle is transporting material extracted during the production process.

The infrastructure variable exhibited the least significance in the data, being under-represented in the interviews. This observation was corroborated during the fieldwork through direct observation ( Figure 5), where it was possible to see adjacent roads improved by the companies themselves to transport their production. This variable is linked to a type of strategy developed by the companies, linked to the relationship with the park authorities – as the companies improve roads and routes within the PNSAC – yet also with the communities living in the park, trying to establish trust with the neighbours by improving and maintaining infrastructures. However, at this point, it is important to highlight the impacts that the transport of production generates on the private property adjacent to the roads, causing structural damage and air pollution, reducing well-being.

In terms of building links with local communities, is one of the most frequently discussed issues in the interviews. However, it is important to note that this issue is of comparable significance in all of the interviews. On the one hand, there are tensions between the uses that each group of actors intends to make of the territory; for example, companies use the territory for their economic activity. On the other hand, there is cooperation in which companies are linked to park authorities which, in turn, relate to the neighbours of the localities close to the mining operation in order to employ them in the operation. It is important to note that the mining industry in the study area, does not generate a significant number of employment opportunities due to the integration of advanced technology in the production process. On average, these companies employ between five and twenty people.

The environmental recovery variable has a greater relative weight in one of the interviews, with only one company reporting the repair of the environmental damages caused during the production process. This is justified by the way in which the damaged area is recovered, which is through the purchase of abandoned quarries from former extractives companies used for the deposit of waste and through reforestation and the water reuse.

A comparable scenario is observed in the environmental impact variable, though, in contrast to the recovery variable, the impact is not mentioned in two of the five interviews and only one of the three remaining interviews explicitly articulates the activity's environmental impact.

With respect to conflict, it emerges as the variable most frequently present in the analyzed data, though it does not possess a heightened relative weight across all interviews. This variable was made explicit in two of the testimonies, and in the rest, it can be found implicitly in the accounts of the interviewed actors. This situation shows that there are tensions surrounding natural resources use in the area under study.

In relation to the variable type of material, this demonstrates the manner in which mining is conducted in and around the park, employing an open pit mining technique with the use of explosive charges to create the initial excavation. This is followed by the implementation of diamond wire and water to exert pressure on the cut walls, resulting in the disintegration of the rock blocks and their subsequent transportation to the milling stage or direct sale in the market.

The data obtained were found to be useful in providing concise descriptions of the phenomena discovered. Finally, the coded segments in the interviews revealed the existing conflicts in the area covered by the natural park, but also revealed the conflicts that took place on the park's borders, i.e. "outer boundaries".

## Conclusions

Since the creation of the PNSAC, a variety of economic activities continued its activity, with quarrying being a prominent landscape feature of territorial configuration. This industry is associated with land clearing and the construction of road infrastructure, resulting in significant territorial transformations that bear serious environmental consequences. Through this configuration, we were able to commence the identification of incipient socio-territorial conflicts within a natural protected area concerning the use and appropriation of land and water.

The D´Aire e Candeeiros case proved to be a particularly notable example, as it demonstrated the evolution of conflicts within a protected area characterized by stringent conservation regulations, yet concurrently allowing for the development of extractive activities that infringe upon the environment and the well-being of local populations. Through the voices of the interviewed individuals, we were able to discern the relationships and strategies employed by these actors. It is evident that companies contribute to the creation and maintenance of PNSAC infrastructures, while the PNSAC facilitates the expansion of mining areas, thereby enabling the ongoing development of mining activities. It is noteworthy that, although this study was based on two of the actors involved in socio-territorial conflicts (companies and PNSAC authorities), the presence of other groups of actors is by no means unknown. This is a striking point for future research, which should include such groups in order to identify the strategies that are being developed.

## Research ethics

We state that we have carried out research in Serra de Aire e Candeeiros, Portugal, within the framework of the HIGHLANDS.3 project activities financed by the European Commission through MSCA-RISE-2019 – Marie Sklodowska-Curie Research and Innovation Staff Exchange grant agreement N. 872328 as part of the program Horizon 2020-EU.1.3.3. –

We further confirm that any aspect of the work covered in this manuscript that has involved humans has been conducted with the ethical approval of all relevant bodies and that such approvals are acknowledged within the manuscript.

The fieldwork was developed using qualitative techniques that included direct observation, field records, and semi-structured interviews with various local actors linked to the object of study. All activities were carried out respecting the ethical principles of social research, including the informed oral consent of the interviewees.

We also declare that the field collections have been carried out by us and form part of the empirical material supporting the research findings. Transcripts of the interviews may be made available for publication in part or in full, depending on the criteria established by the research. Still, they should not be made public due to ethical considerations and the commitment to the participants regarding the confidentiality of the information provided.

## Data Availability

Interviews on socio-territorial conflicts in Serra de Aire e Candeeiros (Portugal). DOI: 10.17632/7p9v22dxy5.1 ^23^. This project contains the following underlying data: Semi-structured interviews and coding tables generated from their analysis were collected to study socio-territorial conflicts between extractive activities and the conservation of a protected natural area. Data are available under the terms of the Creative Commons Attribution 4.0 International license (CC-BY 4.0).
