# Quarry mining or nature conservation, emerging conflicts in Serra de Aire e Candeeiros Natural Park (Portugal)

**DOI:** 10.12688/openreseurope.19589.2

**Published:** 2026-04-16

**Authors:** María Guillermina Diaz, Luis Carreira dos Santos

**Affiliations:** 1 Instituto Argentino de Nivologia Glaciologia y Ciencias Ambientales, Mendoza, Mendoza Province, 5500, Argentina; 2 Instituto Politecnico de Tomar Escola Superior de Tecnologia de Tomar, Tomar, Santarém District, 2300-313, Portugal

**Keywords:** Quarry mining, environmental impact, socio-territorial conflicts, PNSAC, natural resources

## Abstract

This exploratory study examines the socio-territorial conflicts arising from the competition between quarry mining and nature conservation in the Serra de Aire e Candeeiros Natural Park in Portugal. The study utilises content analysis of semi-structured interviews with stakeholders and the snowball technique to estimate the population size and understand the relationships between actors. The findings reveal tensions between mining companies, park authorities, and local communities, resulting in socio-territorial conflicts in the protected area. The study highlights the need for clear outcomes from local development initiatives and the challenges of reconciling mining and conservation in land-use planning policies. This research contributes to the literature gap on non-metalliferous mining activities and environmental conflicts in the study area. Overall, it emphasises the unequal distribution of social and environmental costs associated with extractive activities, underscoring the importance of understanding the dynamics that influence socio-territorial conflicts in highly protected areas.

## Key findings include

***Land Use Conflicts:*** There are clear disagreements over how the land should be used, with mining companies focused on extraction and park authorities on conservation.

***Limited Local Benefits:*** Despite the economic activity, mining doesn’t create many local jobs due to advanced machinery, and local communities often don’t see significant direct benefits.

***Unequal Burdens:*** The environmental and social costs of mining (like pollution and damage to private property, mainly from transport) are often borne by local communities, while the benefits are exported internationally.

***Complex Relationships:*** There’s a complicated relationship where mining companies sometimes contribute to road improvements within the park, and the park, in turn, facilitates the expansion of mining areas.

## What this means for the future

This research highlights the urgent need for better ways to manage conflicts in protected areas. It suggests that public policies should aim to balance economic interests with conservation efforts, while also addressing the inequalities faced by local communities.

The findings show that participatory land use planning involving the entire community is essential to protect natural areas such as the Serra de Aire e Candeeiros Natural Park for future generations.

## Introduction

In Portugal, non-metalliferous mining operations are exclusively conducted by national enterprises. Notably around 90% of the output is designated for export, with a significant emphasis on the Asian market. In 2021, Portugal’s commercial production of aggregates, cement, lime minerals, ornamental stones , and industrial minerals totalled 66,379,493 tonnes, generating a revenue of €459,632. (10 3€) (DGEG - Statistics of Geological Resources of the DSEF-RG).

Mining activities play a vital role in contributing to the economy, however, it is crucial to acknowledge the substantial environmental and social impacts that mineral extraction can have surrounding communities. Recognising these effects is essential for promoting sustainable practices and ensuring the well-being of affected areas. Especially when the geological distribution of a concentrated mineral represents a substantial number of active or previous exploitations in a each area. Minerals can only be extracted from their natural locations, which are often unsuitable for other land uses. This situation favours quarry mining and promotes significant impacts on the surrounding areas. ^1^ As a result, competition for land is produced, which is further reinforced by the selection of sites for extractive activities. Several factors, including resource availability, costs, and socio-economic and environmental considerations are key to its expansion. ^2^

The intersection of mining, an activity that inherently alters landscapes, with conservation, which aims to preserve or restore sustainable natural systems, poses a challenge for environmental and land-use planning policies. ^3^

The current European legal and regulatory framework for environmental protection allows mining activities in natural protected areas. The European Commission states that there is no automatic ban on Non-Energy Extractive (NEE) industry activities within or near the Natura 2000 network. Mining activities must comply with Article 6 of the Habitats Directive. They require an appropriate assessment to ensure that they do not harm the integrity of Natura 2000 sites. Additionally, each mining project must be reviewed individually, considering its specific conditions and potential impacts.

The Natural Park (NP) of Serras de Aire e Candeeiros presents a unique situation within the Natura 2000 Network, further classified as a Natural Park. Its primary goal is to protect the natural values of the Extremadura Calcareous Massif, though it also permits the expansion of mining activities by allowing the rehabilitation of abandoned quarry sites as part of the restoration purposes of the NP. In this sense, 4 Machado *et al.* (2014) define a *difficult coexistence* generated between the natural heritage and the aggregate extractive industry that has been developed in the park before its classification.

Forty-five years since the park’s establishment, a thin line continues to separate nature conservation from the growing mining activities. The lack of clear outcomes from local development initiatives is evident, as the ageing population and demographic decline become increasingly noticeable in a region shaped by the economic interests of globalization, open markets, and the depletion of natural resources. While the Sustainable Development Goals (SDGs) offer a new perspective on sustainability for the 21st century, deeply rooted traditions and contradictory approaches to sustainable development may pose significant barriers to achieving these important objectives.

Likewise, the clear contradictions that can be interpreted in the Management Plan for the Serra de Aires e Candeeiros Natural Park (POPNSAC) allow us to frame the clash of interests in the area under study, which reveals the environmental conflict generated by the uses that the different actors make of the territory. From this point of view, we define socio-territorial conflicts as situations in which there is a clash of interests between two or more groups of actors over the use and appropriation of natural resources. At this point, we emphasize that socio-territorial conflicts are primarly social and strategic in nature. These conflicts arise over time, making temporality a key characteristic of their development, as well as influencing the measures that social actors take as the conflict is unfolds. From this definition, the focus is on the unequal spatial distribution of the negative social and environmental costs and impacts that can be caused by the development of extractive activities by one group of actors over others. ^5– 9^

This study highlights the significance of the socio-environmental implications associated with non-metallic mining, which frequently serve as the basis for disputes. Consequently, during the data analysis, we approached these implications as an integrated whole, alongside a thorough examination of the conflicts present in the study area. In this regard, how is the territory restructured by the development of extractive activity in a classified protected area? What influence does territorial configuration have on the emergence of environmental conflicts? These questions serve as a guide for this study, which aims to identify the presence of socio-territorial conflicts in a highly protected area.

## Literature review

Recent academic literature indicates that environmental conflicts related to mining activities in central Portugal can be traced back to the 19th century. As mentioned by 10 the cases of Braçal (1836–1959) and Talhadas (1889–1931), with the exploitation of Copper, Silver, and Lead, other Portuguese regions with lesser mediatic impact were also identified. The same authors unveil mining conflicts in places such as the Algarve: Tavira (Santa Catarina da Fonte do Bispo), Monchique (Carapitotas, Corte Grande); North: Barcelos (Barqueiros, Vila Seca and Milhazes); Center: Coimbra (Cantanhede, Soure, and Mira), Figueira da Foz (Ferreira-a-Nova and Bom Sucesso), Leiria (Pombal), Viseu (18 Municipalities), Sever do Vouga (Braçal mining complex); Águeda (Talhadas mines); Alentejo: Évora (Boa-fé, Serra de Monfurado), Portalegre (Nisa). Using a methodology that combines documentary literature and social analysis, the authors report significant social mobilization and protests that have influenced current legislation, ^11^ and analyse the socio-environmental conflicts occurring in Portugal between 1975 and 2015. They examine various factors, including the level of mobilization, media attention, organization, impact, and ideological references that influenced these conflicts during this period.

At the local level, Serra de Aires e Candeeiros has been the focus of numerous studies concerning mining activities. However, these investigations have generally revealed minimal to on association with environmental conflict. Most research emphasizes the connection between mining operations and economic development. In this sense, 12 most studies carried out a detailed analysis of the geological conditions of the study area, highlighting its potential for the exploitation of ornamental limestone, claiming it would bring important benefits to regional development.

It is important to highlight that the land-use planning policy for the Serras de Aire e Candeerios Natural Park facilitates the expansion and operation of existing companies. This is achieved by granting new licenses to individuals who committo implementing decommissioned quarry restoration practices. With that in mind, some authors. ^12, 13^ proposed a methodology to define and delimit the associated geological suitability areas, aiming to reduce environmental conflicts in the Serras de Aire e Candeerios Natural Park by integrating parks’ objectives.

Ferreira Carvalho *et al..* ^13^ also identified five nuclei, Moleanos, Salgueiras, Cabeca, Veada, Pé da Predeira and Codacal, where ornamental stones quarrying is carried out in the Natura 2000 Network area within the NP. Concomitantly, the authors highlight the existing problems linked to the compatibility of mining activity with natural environment conservation, as do the actors involved in the process, whom they define as those responsible for the territorial management and knowledge, the public sector, and the private sector (mining companies and their associated entities).

Other studies also reveal the connection between mining activities in the NP and the development opportunities this industry can bring to local communities. By doing so, 14 using a geological approach, carried out a study to unravel the questions linked to the possibility of developing extractive activity of the mineral masses compatible with local development, that is, to think about the possibility of added value that quarries could bring to territories in which they are located. The author seeks to contribute to the identification of the challenges that mining operations represent for local territorial planning and development.

Upon reviewing the literature, we were able to highlight the environmental issues and conflicts arising from mining activities at both national and local levels. These conflicts often emerges between mining operations and communities living in protected areas, such as those within the PNSAC. However, most of the cited literature primarily emphasizes local development related to conflict surrounding metalliferous mining, with considerably less attention given to quarry mining.

Additionally, the aforementioned works highlight a gap in literature regarding non-metalliferous mining activities and environmental conflicts, especially in the PNSAC area. This paper seeks to make a meaningful contribution towards addressing this gap.

## Study area

Portugal is located on the Iberian Peninsula, covering an area of 92,061 km², which includes a continental part measuring 88,944 km². The Extremadura Calcareous Massif (ECM) spans an area of 900 km² in central Portugal, approximately 100 km from Lisbon. This massif consists of a thick sequence of Mesozoic carbonate rocks that are structurally elevated. The lithostratigraphy of the area is well understood, with primary ornamental rock varieties dating back to the Middle Jurassic period, specifically the Batonian and Calovian ages. The ECM has five main locations where extractive activities occur: the Pé de Pedreira, Moleanos, Codacal, Fátima, and Alvados areas.

The highlands of this limestone massif are classified as a natural protected area comprising the D’Aire and Candeeiros mountains, which hold most of the extractive industry.

**Figure 1.**
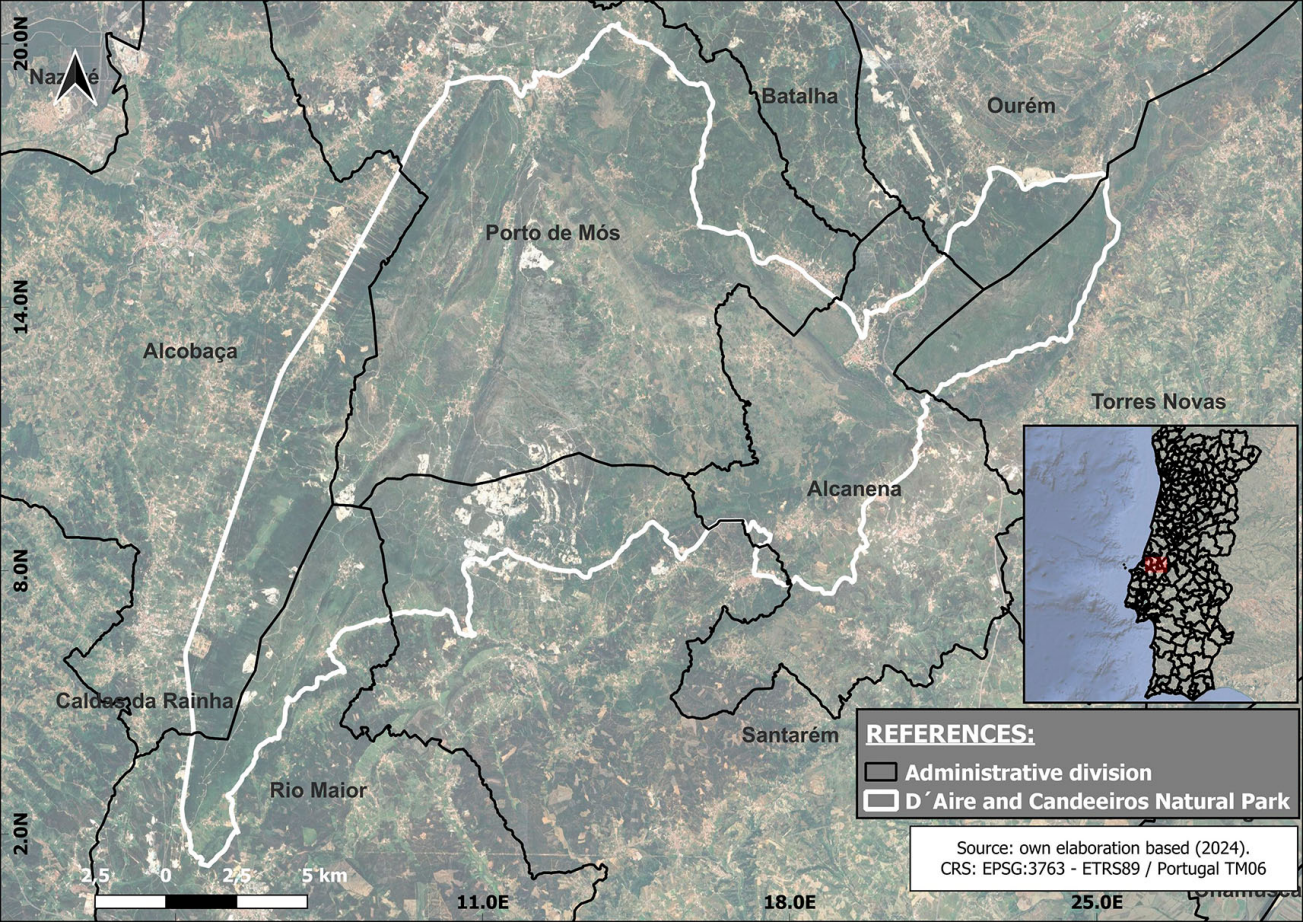
Geographical location map. The black lines show the administrative division of the region under study, and the white lines delimit the area comprising the Serra D'Aires e Candeerios Natural Park.

The area is administratively part of the Santarém and Leiria Districts and includes seven municipalities. In the Santarém District, the municipalities are Alcanena, Rio Maior, Santarém and Torres Novas. In the Leiria District, the municipalities are Ourém, Alcobaça, and Porto de Mós ( Figure 1). The NP of Serras D’Aire e Candeeiros is one of the 13 Natural Parks of Portugal. The NP of Serras de Aire e Candeeiros was declared in 1979 by decree-law (N°18/79) with the main objective of safeguarding the natural and heritage values of the ECM, also holding classified areas by the Natura 2000 Network.

Located in the centre-west of Portugal and covering an area of approximately 38.900 ha the D’Aire and Candeeiros mountain are the most important reservoir of limestone formations in Portugal, their karstic morphology, the nature of the vegetation cover, the network of underground caves and watercourses, the endemic fauna and flora are the main aspects that contributed towards the classification of this area as a NP. Nonetheless, the abundant limestone natural resources undergo an intense pressure from the activity in the field of mineral resource extraction.

In terms of orographic features, four zones can be distinguished: two mountain ranges, the D’Aire (678 m) and the Candeeiros (615 m), and two plateaus, the Santo António and the São Mamede. ^15^ There are numerous underground karst formations and some notable features, such as the *poldje* of Aire-Minde, Mira, and Mendiga dolines. Lastly, the park also features several landforms that result from the dissolution of rock due to water infiltration through diaclases.

Climatically, 15 states that Portugal’s climate is mainly temperate Mediterranean. The peculiar characteristics of the Park’s microclimate are: first, the thermometric characteristics, the average annual temperature registers values between 13°c and 15°c. The high summer temperatures are softened or tempered by the proximity of the Atlantic Ocean, resulting in July being the warmest month, with an average temperature varying between 18° and 24°C. The significant winter rainfall deserves attention, with annual averages ranging from 800 to 1,200 mm.

Regarding the area’s biogeography, 15 notes that the vegetation defining the PNSAC stems from the Mediterranean bioclimatic influences on the region, predominantly rocky areas with lush vegetation on limestone summits, slopes with tree cover, scrubland, undergrowth (garrigue), and orchards found in depressions.

The aforementioned natural attributes are conducive to developing agricultural and livestock activities located in the main fertile urbanized depressions.

In demographic terms, according to data from the Portuguese National Institute of Statistics [1], by 2021 the park’s municipalities approximately 20,000 inhabitants, mainly in the valleys surrounding the Sierras de Aire and Candeeiros and below the plain of San Antonio. This accounts for the scarce presence of a stable population living within the Park.

## Method

The nature of this research compelled a specific methodological design consisting of three stages:

1- The first stage involved the delimitation of the study area and the definition of the subject participants.
2- The second stage, considered the search for extractive companies operating in the region and data collection (semi-structured interviews).
3- Finally, the third stage consisted of data sorting and analysis.

The initial exploration of the study area involved a review of technical literature. This included examining various documents, such as the Territorial Management Plan of the Serra de Aire e Candeeiros Natural Park, as well as searching for official statistical data published by the Portuguese General Directorate of Energy and Geology (DGEG).

Georeferenced information was utilized to create a map of the total area encompassing the PNSAC, the mining zones within the park and its surrounding boundaries. The mapping was conducted using QGIS version 3.26.1 Buenos Aires. This process successfully identified the mining areas within the park, indicating that the fieldwork will take place in several municipalities: Alcanena, Alcobaça, Ourém, Porto de Mós, Rio Maior, Santarém, and Torres Novas ( Figure 1).

Once the area was established, contacts were made with extractive companies, considering those willing to provide information on possible socio-territorial conflicts and environmental issues in the geographical study area. Based on the delimitation of the study area, the mining companies operating within the PNSAC were selected.

It should be noted that when working with a population for which there is insufficient information to define a representative sample, the snowball technique was used to estimate population size and understand central aspects such as the types of links they have with each other. ^16^

Extractive activity, as a business, is bound by hermeticity and competition in this economic sector. As stated in the existing literature, the population was defined as hard-to-reach populations. ^17– 20^

The term hard-to-reach population refers particularly to populations that do not wish to be contacted or identified based on specific criteria, as well as groups that are considered difficult to reach because of their geographical location. 16, 20 define the groups of people who are sometimes considered difficult to reach or hidden, as those subjects who have no interest in being found or contacted.

The authors state that “In these circumstances, it can be very difficult or expensive to recruit study subjects” recommending the use of snowball sampling method as a reliable referrals-based method, indicating that “initial known subjects to recruit new additional subjects.”. ^20^

Accordingly, 21 argue that a chain-referral sampling method relies on referrals from initial subjects to generate additional subjects.

**Figure 2.**
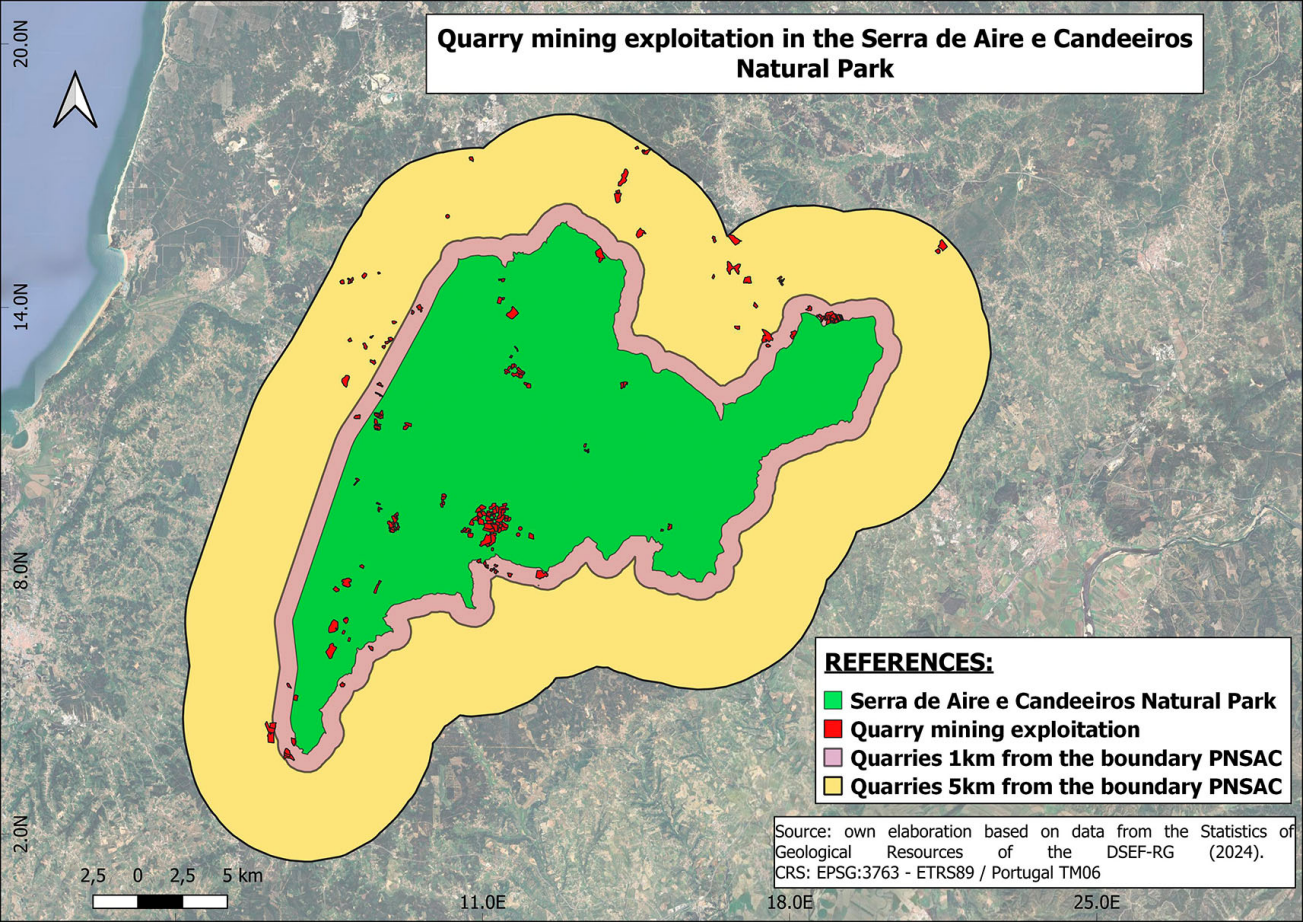
Location of mining operations. The area covered by the Serra de Aires e Candeeiros natural park is shown in green. The red colour shows all the mining operations that operate within the park and its borders. The purple colour shows the distance between the boundary of the park and the mining operations within a distance of 1 km. The yellow colour represents the distance between the park boundary and the mining operations within a distance of 5 km.

The criteria used to identify key stakeholders and thus form the sample were: first, companies must operate within the PNSAC, i.e. the selected mining company must be located within the park or within a radius of 1 km to 5 km from of the park ( Figure 2); the second criterion relates to the size of the quarry, between 1 and 15 ha, with a quarry of 1 ha being defined as small and one of 15 ha or more as large . The third criterion is the distance to a population centre of up to 5 km ( Figure 2).

Quarry mining data was resampled from the DGEG Portuguese National database using QGIS to select mining companies within the set boundaries. Resulting in 195 quarries selected, considering both 1km and 5km buffers. Where 39 quarries are located within the 1km buffer, 38 are located between the 1km and the 5km buffer, and 118 quarries are located inside the NP.

**Figure 3.**
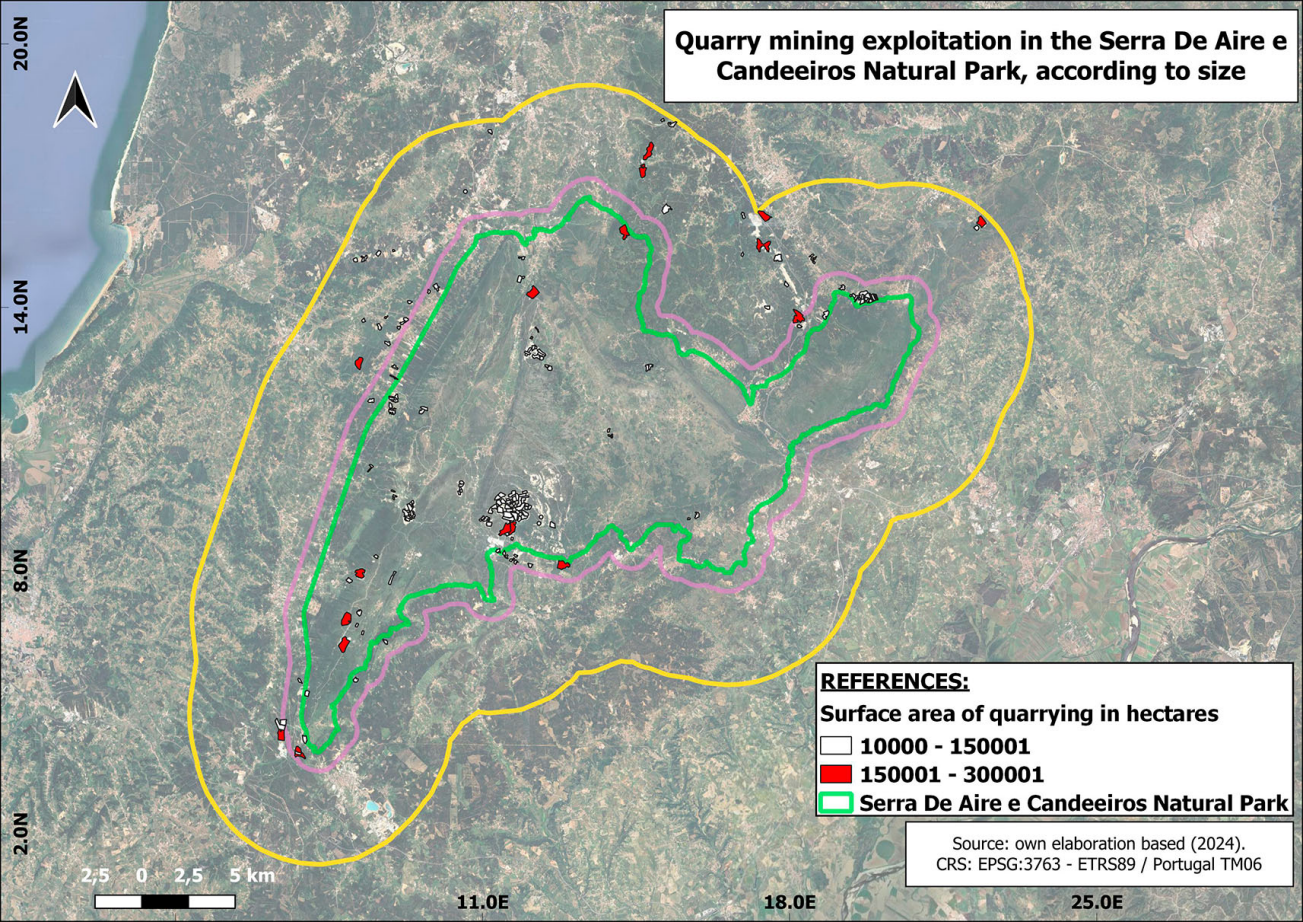
Location of mining sites according to their size. In white colour are represented the mining exploitations that have a surface size between 1 and 15 hectares. In red colour are represented the mines with a surface between 15 and 30 hectares. The green perimeter represents the boundary of the natural park.

In terms of the size of the selected companies, 18 large quarries and 117 small ones were identified. ( Figure 3)

Despite the use of systematic sampling, the snowball technique was used to select the companies to be interviewed. This technique allowed us to identify only 4 quarries as representative samples for the study. Of the five interviews, four were conducted with mining companies within the park and one with Natural Park staff.

In this way, 5 semi-structured interviews were carried out, which aimed to gather information on the activity developed by the quarries, specifically their links with other actors in the area where they operate, without neglecting to mention those issues that brought us closer to the identification of socio-territorial conflicts and the environmental impacts generated by the activity.

Ethical clearance was granted through the Highlands.3 project under European Union Horizon 2020 regulations, ensuring compliance with international research standards. All respondents received a written and oral description of the project in the local language and verbal/recorded informed consent forms. We emphasised to all participants our commitment to the principles of confidentiality and respect for cultural and institutional sensitivities.

To complete the data collection, semi-structured interviews were designed and conducted with the 4 selected mining companies and, in parallel, direct field observation was carried out.

The study area was visited for interviews, and visual data, namely photographs and videos, were collected, generating a valuable photographic and film archive. The analytical framework applied combines thematic content analysis with principles of systematic qualitative coding. First, all semi-structured interviews were recorded and transcribed verbatim; the transcripts were checked against the recordings to ensure accuracy. The initial phase consisted of repeated readings of the transcripts for familiarisation and the generation of analytical memos. Coding was performed in two steps: (1) open and inductive coding to identify meaningful units of text (relevant phrases, paragraphs, and quotations) and (2) axial/deductive coding to relate codes to pre-existing theoretical categories where appropriate. In this way we develop a codebook defining each code, inclusion/exclusion criteria, and textual examples; this codebook was updated iteratively as new codes emerged.

## Results and analysis

Based on the interview questions, a list of 9 codes was generated ( Table 1) with their own definitions, allowing the methodological variables to be analyzed to be specified.

**Table 1.** Code definitions. Own elaboration, 2023.

Code	Definition
**Conflict**	Confrontation of interests between different actors in the use of natural resources.
**Environmental Impact**	Water contamination, landscape deterioration, land clearing, pollution.
**Local community**	Liaison with the neighbours of the localities surrounding the quarrying area.
**Production process**	Refers to the form of material extraction.
**Infrastructure**	Construction or maintenance of roads.
**Type of material**	Rocks extracted in the production process
**Market**	To where the production is marketed, i.e. to the international or national market.
**Natural Park**	Existence of links between extractive companies and the park.
**Environmental recovery**	Set of actions carried out by extractive companies to remediate or restore the natural conditions of an environmentally degraded site.

The coding system was created inductively, through the analysis of interview content. This system contains a total of 9 codes ( Table 2). The data analysis was carried out in MaxQDA, through this software the codes in the interviews were identified in order to analyze the qualitative data.

**Table 2.** Coding system resulting from interviews.

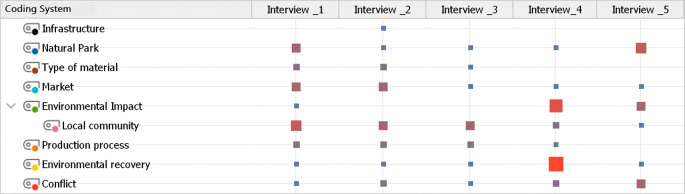

Table 2 shows the weight of each code analyzed in the different interviews, and then the *word count function* and the *sum* were used to analyze the preponderance of the codes in each document.

The utilisation of the devised coding system enables the observation of the relative weight of each code in the interviews. The number of instances of the codes in the documents was considered to ascertain this relative weight. Of the nine codes identified, five have a higher weight, which is represented in the table with a larger size and in dark red: local community, environmental remediation, environmental impact, conflict, and type of material. Conversely, the smaller quadrants indicate a lower relative weight linked to a low presence of the codes in the interviews.

It should be noted that upon the quantification of all summed analyzed variables, the number of variable occurrences in the interviews was calculated for each variable and code. This resulted in a discrepancy between the documents, as some presented a greater number of variables identified in the accounts of the interviewed actors. Additionally, a discrepancy was observed in the coding system itself, where the resulting values allowed for the observation of differences in their frequency in the interviews.

**Figure 4.**
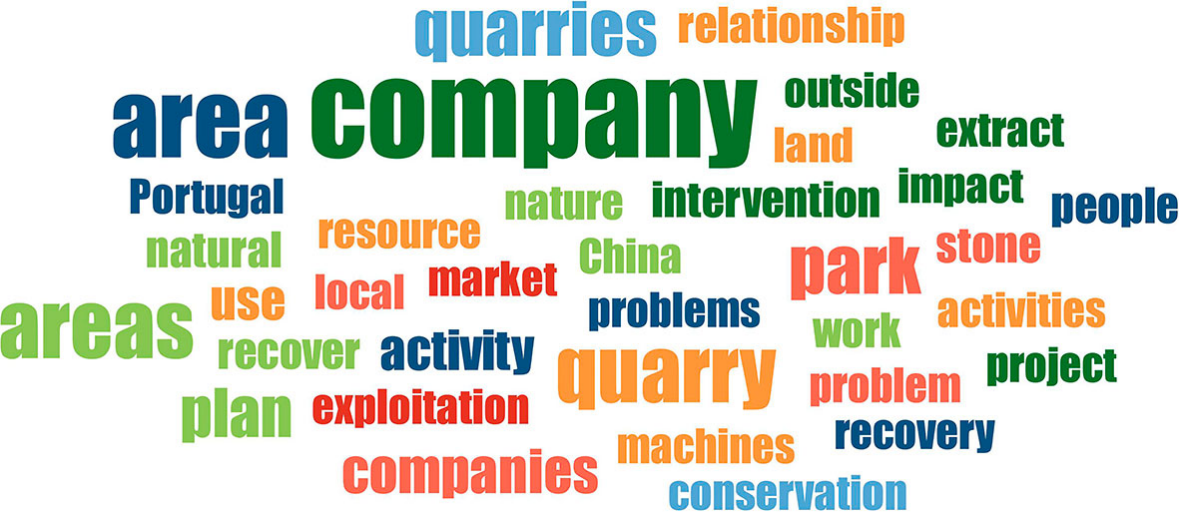
Word cloud created from the thirty-five most representative words obtained from the interviews. The colours are random.

In the context of word frequency analysis, the objective of this task is to ascertain the terms that are most frequently cited by the interviewed subjects, with a view to identify those that are of the greatest pertinence. For word frequency count, the fifty most prevalent words were designated as the selection criteria. The list was subjected to three iterations of purification, during which the so-called ‘empty words’ or words devoid of content were discarded. The following word cloud ( Figure 4) illustrates the thirty-five most representative words (with the highest percentage load).

The word cloud serves to illustrate the presence of companies operating within and outside the PNSAC, and the nature of their activities. Concerning the nature of these activities, quarrying is recognized as an extractive industry, carried out with the use of state-of-the-art machinery and technology to exploit resources, which is associated with the absence of employment for the local communities living in the PNSAC. The word cloud provides a clear illustration of the market(s) to which the production is destined, namely the international and national markets, with exports destined for China and the remainder distributed within Portugal.

Despite the fact that the companies undertake remediation initiatives in areas of exploitation through reforestation or water reuse projects, as evidenced by one of the companies, the word cloud underscores the environmental implications that this type of activity can engender in a protected area through the intervention that is made on its territory. This is further compounded by the challenges that the development of mining engenders in terms of land use among the various stakeholders residing there, giving rise to socio-territorial conflicts.

The challenges posed by mining development significantly affect land use among various stakeholders in the area, leading to socio-territorial conflicts. For instance, disputes often arise between neighbours and companies due to the dust and explosions generated by daily blasting activities in the mines. Additionally, the heavy water consumption by companies for washing the extracted materials has further highlighted conflicts in the region.

These issues contribute to the socio-environmental impacts of quarry mining. Specifically, the dust from blasting settles on the vegetation surrounding the mines, causing harm. Regarding water usage, the most significant impact is the excessive consumption by companies, which limits the supply available to the communities neighboring the park.

## Discussions

The analysis indicates that the conflicts stem from a specific type of land use: open-pit quarrying. This method primarily targets ornamental stones and limestone through a cutting technique that utilizes water in the extraction process to meet the demands of an international market. Community engagement in the surrounding areas is minimal, and the nature of the quarrying operation limits employment opportunities. The production process predominantly relies on mechanised solutions, resulting in a reduce number of jobs.

The relationship with the natural park involves the issuance of exploitation permits focused on the reclamation ofabandoned quarries that existed prior to the park’s establishment. Mining companies perceive this as an opportunity to access new locations while responsibly depositing waste rock in these quarries. They assert that this approach will facilitate recovery efforts and foster the development of a lucrative export business for high-quality materials. The park benefits from a maintained access network, while the government gains tax revenue from the company’s operations.

A market analysis revealed that companies export 90% of their production, which leaves only a small margin for sales and distribution within the Portuguese national market. The primary buyers of marble blocks extracted by these companies are located in Asia, while the leftover material from the production process is intended for de domestic market.

**Figure 5.**
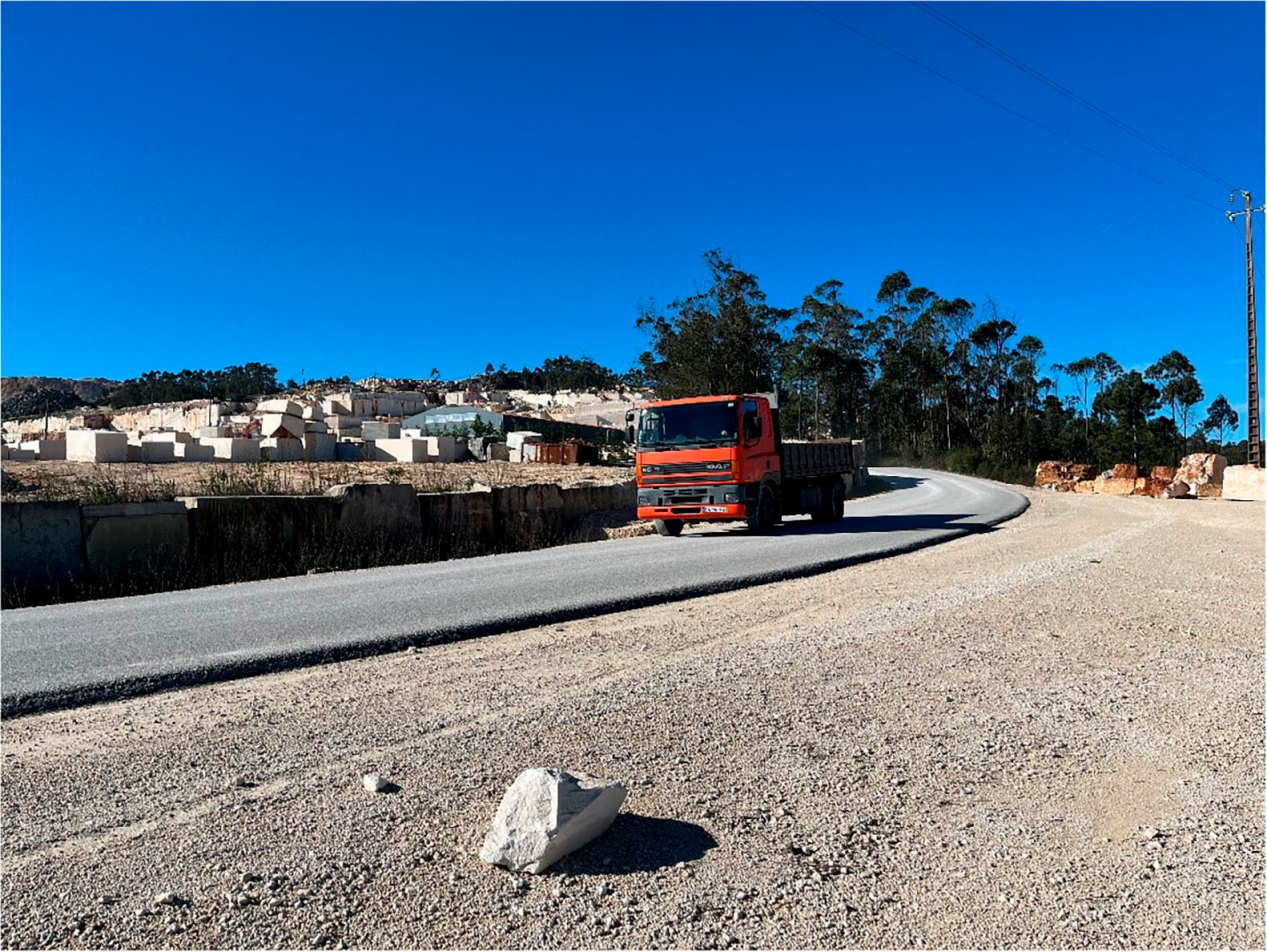
Photograph taken during fieldwork on one of the visits to the mining sites. The vehicle is transporting material extracted during the production process.

The infrastructure variable exhibited the least significance in the data, being under-represented in the interviews. This observation was corroborated during the fieldwork through direct observation ( Figure 5), where it was possible to see adjacent roads improved by the companies themselves to transport their production. This variable is associated with a strategy developed by the companies that focuses on their relationship with the park authorities. These companies improve roads and routes within the PNSAC. Additionally, they aim to build trust with the communities living near the park by enhancing and maintaining local infrastructure. However, it is important to highlight the negative impacts of transporting production. This transport can cause structural damage to private properties adjacent to the roads and lead to air pollution, ultimately reducing the well-being of residents.

The importance of establishing connections with local communities is a recurring theme in our discussions and holds considerable significance across all interviews conducted. There are inherent tensions among various stakeholders regarding their intended use of the territory; for instance, companies utilise the land primarily for economic activities. Conversely, collaboration exists where companies engage with park authorities, who then reach out to local residents to reassure them regarding mining operations. It is important to acknowledge that the mining industry within the study area does not create a significant number of job opportunities. This is primarily due to the incorporation of advanced technology within the production process. Typically, these companies employ between five and twenty individuals, which is adequate for a small family-run business up to larger enterprises operating across multiple extraction sites.

The environmental recovery variable has a greater relative weight in one of the interviews, with only one company reporting the repair of the environmental damages caused during the production process. This is justified by the way in which the damaged area is recovered, which is through the purchase of abandoned quarries from former extractives companies used for the deposit of waste and through reforestation and the water reuse.

The environmental impact data shows a similar pattern, but unlike the recovery data, two out of five interviews don’t discuss the impact, and only one of the three remaining interviews directly addresses the environmental consequences of the activity. Leading us to believe that there is no assurance that they intend to rehabilitate beyond the operational period, abandoning the new quarry and leaving the inherited environmental consequences.

With respect to conflict, it emerges as the variable most frequently present in the analysed data, though it does not possess a heightened relative weight across all interviews. This variable was made explicit in two of the testimonies, and in the rest, it can be found implicitly in the accounts of the interviewed actors. This situation illustrates the tensions surrounding natural resources in the studied area, indicating a certain degree of expressed sentiment, as the legal framework favours the companies. Additionally, social contributions that have minor economic significance for the companies are often evident, such as local festivals.

In relation to the variable type of material, this demonstrates the manner in which mining is conducted in and around the park, employing an open pit mining technique with the use of explosive charges to create the initial excavation. This is followed by the implementation of diamond wire and water to exert pressure on the cut walls, resulting in the disintegration of the rock, cutting blocks in benches and their subsequent transportation to the milling stage or direct sale in the market.

Given the exploratory and qualitative nature of this research, the small number of interviews (five) constitutes an important limitation that constrains the extent to which the results can be generalised to all actors and conflicts in PNSAC. The perspectives captured here primarily reflect specific configurations of relationships between quarry companies and park authorities, and therefore offer an indicative rather than exhaustive picture of the socio territorial dynamics at stake. This is consistent with broader reflections on mining and quarrying as land use processes marked by spatial constraints, path dependency and significant uncertainty regarding long term trajectories, which make it difficult for any single study to fully encompass the range of impacts and future land use options associated with extractive activities (Bloodworth *et al.*, 2009). Consequently, future research should expand the number and diversity of interviewed stakeholders, including local residents and other public and civil society actors, to deepen understanding of how quarrying reshapes territorial futures in protected areas.

The data obtained were found to be useful in providing concise descriptions of the phenomena discovered. Finally, the coded segments in the interviews revealed the existing conflicts in the area covered by the natural park, but also revealed the conflicts that took place on the park’s borders, i.e. “outer boundaries”.

## Conclusions

Since the establishment of the PNSAC, a range of economic activities has continued to take place, with quarrying being a prominent landscape feature of territorial configuration. This industry is associated with land clearing and the construction of road infrastructure, resulting in significant territorial transformations that bear serious environmental consequences. Through this configuration, we were able to commence the identification of incipient socio-territorial conflicts within a natural protected area concerning the use and appropriation of land and water.

The D’Aire e Candeeiros case proved to be a particularly notable example, as it demonstrated the evolution of conflicts within a protected area characterized by stringent conservation regulations, yet concurrently allowing for the development of extractive activities that infringe upon the environment and the well-being of local populations. Through the voices of the interviewed individuals, we were able to discern the relationships and strategies employed by these actors. Companies contribute to the creation and maintenance of PNSAC infrastructures, while the PNSAC facilitates the expansion of mining areas, thereby enabling the ongoing development of mining activities. It is noteworthy that, although this study was based on two of the actors involved in socio-territorial conflicts (companies and PNSAC authorities), the presence of other groups of actors is by no means unknown. This is a striking point for future research, which should include such groups in order to identify the strategies that are being developed.

## Research ethics

We state that we have carried out research in Serra de Aire e Candeeiros, Portugal, within the framework of the HIGHLANDS.3 project activities financed by the European Commission through MSCA-RISE-2019 – Marie Sklodowska-Curie Research and Innovation Staff Exchange grant agreement N. 872328 as part of the program Horizon 2020-EU.1.3.3. –

We further confirm that any aspect of the work covered in this manuscript that has involved humans has been conducted with the ethical approval of all relevant bodies and that such approvals are acknowledged within the manuscript.

The fieldwork was developed using qualitative techniques that included direct observation, field records, and semi-structured interviews with various local actors linked to the object of study. All activities were carried out respecting the ethical principles of social research, including the informed oral consent of the interviewees.

We also declare that the field collections have been carried out by us and form part of the empirical material supporting the research findings. Transcripts of the interviews may be made available for publication in part or in full, depending on the criteria established by the research. Still, they should not be made public due to ethical considerations and the commitment to the participants regarding the confidentiality of the information provided.

## Data Availability

Interviews on socio-territorial conflicts in Serra de Aire e Candeeiros (Portugal). DOI: 10.17632/7p9v22dxy5.1. ^22^ This project contains the following underlying data: Semi-structured interviews and coding tables generated from their analysis were collected to study socio-territorial conflicts between extractive activities and the conservation of a protected natural area. Data are available under the terms of the Creative Commons Attribution 4.0 International license (CC-BY 4.0).

## Footnotes

^1^
INE. https://censos.ine.pt/xportal/xmain?xpgid=censos21_produtos&xpid=CENSOS21&xlang=pt#op_geocen
