# Supplementary material for: Quarry mining or nature conservation, emerging conflicts in Serra de Aire e Candeeiros Natural Park (Portugal)
Source: Open Res Eur. 2026 Apr 16;5:348. Originally published 2025 Nov 11. [Version 2] doi: 10.12688/openreseurope.19589.2 (PMC13153768; doi:10.12688/openreseurope.19589.2)
Supplement: Peer review history

# Reviewer report: Reviewer response for version 2

**DOI:** 10.21956/openreseurope.24451.r73408

**Published:** 2026-05-20

**Authors:** Isiaka Adio Hassan

**Affiliations:** 1 Lagos State University of Science and Technology, Lagos State, Nigeria

ABSTRACT :

 It is poorly written 

 (1) Sample size not mentioned

 ( 2) Statistical tool used e.g Hypothesis tested not mentioned

 (3) Some statements that should not be found in the Abstract were included e.g." This research contributes to the literature gap on non-metalliferous mining activities and environmental conflicts in the study area" etc.

 The abstract should contain the following in brief, not more than one to three lines of sentence

 (i) Statement problem

 (ii) Aim and objective ( theme of the paper)

 (iii) Description of Method used: to collect samples or information, sample size collected from the population, method adopted to carry out the Laboratory analysis or otherwise on the sample collected.

 Statistical data analysis: Mentioning of statistical tools used to analyse data collected or generated from samples or information collected from the field. e.g descriptive or inferential statistics (hypothses testing)

 Result: Only salient results that could draw attention, not all the results generated from the data 

 Conclusion: This is an inference drawn from your result, that is whether your result is positive to aim and objective or not

 Recommendation: it is not compulsory, provided there is space for the number of words stipulated by journal, In addition, it must not be more than one line sentence

 INTRODUCTION

 The introduction is okay

 LITERATURE REVIEW

 Is not adequate and too local, that is, most reviewed literatures are on Portugal, there are many literatures to review online that can give real research gap. What the author(s) termed as a gap. It is not really a gap, as there are many works on it internationally. It is a common occurrence among the host communities and quarry operators. Which have been addressed by many authors.  Probably, the author(s) are trying to domesticate the gap to Portugal,

 METHODOLOGY

 Is okay; only there is no statistical hypothesIs test to validate the results obtained from the data collected in the field.

 RESULT AND DISCUSSION

 However, manners of the results and discussion presentation are okay. Only that the basis of the result and discussion is faulty, as the results were not properly generated as mentioned earlier; hence, it could not be true reflection of the situation in the field 

 REFERENCE 

 The reference style of the journal was not strictly follow. There are various styles used by author (s), e.g. Vancouver, APA style etc.

 GENERAL REMARK:

 In addition, if all these aforementions are faulty,  It means the manuscript need thorough major correction.

If applicable, is the statistical analysis and its interpretation appropriate?

No

Is the study design appropriate and is the work technically sound?

Yes

Is the work clearly and accurately presented and does it engage with the current literature?

Partly

Are the conclusions drawn adequately supported by the results?

No

Are sufficient details of methods and analysis provided to allow replication by others?

Yes

Are all the source data and materials underlying the results available?

Yes

Reviewer Expertise:

Environmental management, ecotoxicology and Environmental microbiology

I confirm that I have read this submission and believe that I have an appropriate level of expertise to state that I do not consider it to be of an acceptable scientific standard, for reasons outlined above.

# Reviewer report: Reviewer response for version 2

**DOI:** 10.21956/openreseurope.24451.r72628

**Published:** 2026-05-07

**Authors:** Mohamed Fredj

**Affiliations:** 1 Mining and geology Department, University of Bejaia, Faculty of Technology (Ringgold ID: 210392), Béjaïa, Béjaïa Province, Algeria

The article addresses a topic that is highly relevant for European policy: socio‑territorial conflicts between non‑metalliferous quarrying and nature conservation in a Natura 2000 / Natural Park site (PNSAC, Portugal). It aligns well with EU priorities on sustainable development, resource management, and protected‑area governance.

 The paper presents an exploratory qualitative case study based on semi‑structured interviews with quarry companies and park staff, complemented by spatial analysis (QGIS) and systematic coding (MaxQDA). Its main originality lies in documenting conflicts around quarry activities (non‑metalliferous) in a highly protected area, which is a clear gap in the Portuguese literature.

 Scientifically, the manuscript is promising but constrained by several limitations: a small and skewed sample of actors, lack of direct community voices, and an incomplete integration of the theoretical framework (socio‑territorial conflict, environmental justice) with the empirical analysis. With targeted revisions—clarifying objectives, explicitly discussing methodological limits, strengthening theoretical linkage, and policy implications—the article has good potential.

 1-The title is clear, informative, and accurately reflects the location (PNSAC), sector (quarry mining), and main theme (emerging conflicts).

 2-The revised abstract more clearly presents the background, the methodological approach (interviews, content analysis, snowball sampling, QGIS), the key findings (tensions among companies, park authorities, and communities; unequal distribution of costs), and the general conclusions.

**Points to improve**

 1- Add one explicit sentence in the abstract stating the main objective or research question (e.g., “This paper aims to examine…”).

 2- Specify, in the abstract, the number of interviews (5), the actor groups (4 companies, 1 park staff) and the type of analysis (thematic coding, MaxQDA) to make the methodological scope transparent.

 3- More clearly distinguish, in the abstract, empirical findings (what was observed) from interpretive claims (e.g. about unequal cost distribution or policy contradictions).

**I. Introduction**

 1-Justify more explicitly why PNSAC is a relevant and strategic case: intensity and history of quarrying, dual status (Natura 2000 and Natural Park), specific contradictions within the management plan.

 2-Clarify the conceptual framework:

      - Condense the operational definition of “socio‑territorial conflict” into one clear paragraph.

      - Explicitly link this definition to one or two broader frameworks (political ecology, environmental justice, territorial governance) and state which dimensions will be analysed (distributional, procedural, recognitional).

 3- Conclude the introduction in a more standard structure: (i) identified literature gap, (ii) research questions or objectives, (iii) specific contribution of the paper.

**II. Results & Analysis**

 1- Separate description and interpretation more clearly: some interpretive claims (e.g., about the legal framework favoring companies) appear already in the results and would fit better in the discussion.

 2- Make more analytical use of the word cloud by explicitly relating the co‑occurrence of terms such as “market”, “China”, “export”, “environment”, “community”, and “park” to the notion of global commodity chains intersecting with localized socio‑environmental impacts.

**III. Discussion**

 1- Address “latent” or “low‑visibility” conflict: discuss why open conflict and mobilization appear limited despite high local costs (demographic decline, limited economic dependence on quarry jobs, power asymmetries with firms, weak local organizational capacity).

 2- Analyze more explicitly the socio‑economic implications of very limited job creation (5–20 workers per company) for local bargaining power and social license to operate.

 3- Clarify methodological limitations within the discussion as well: small N, partial actor set (no direct interviews with local residents), possible under‑representation of more contentious community perspectives.

**IV. Conclusion**

 1- Develop a more explicit and structured set of “policy implications”, for example:

 The need for transparent and participatory land‑use planning at PNSAC scale, including local communities in decisions on quarry expansion and rehabilitation.

 2- Clear rehabilitation obligations, independent monitoring, and cumulative impact assessment across multiple quarries rather than project‑by‑project approvals.

 3- The importance of aligning authorisation practices and monitoring with the spirit of Article 6 of the Habitats Directive (appropriate assessment and integrity of Natura 2000 sites).

 4- Briefly articulate how lessons from the PNSAC case may inform the management of other Natura 2000 sites where non‑energy extractive industries operate.

**Minor comments**

 1- Standardise acronyms (PNSAC, NP, ECM, DGEG, SDGs) and consider adding a list of abbreviations.

 2- Ensure that figure legends are fully legible, and that color schemes are suitable for colour‑blind readers (avoid problematic red/green combinations).

 3- Clarify the central analytical role of the word cloud: either integrate it more strongly into the narrative or move it to supplementary material if necessary.

 4- Harmonize terminology (“quarry mining”, “quarrying”, “non‑metalliferous mining”, “ornamental stone quarries”) to improve internal consistency.

If applicable, is the statistical analysis and its interpretation appropriate?

Yes

Is the study design appropriate and is the work technically sound?

Yes

Is the work clearly and accurately presented and does it engage with the current literature?

Yes

Are the conclusions drawn adequately supported by the results?

Yes

Are sufficient details of methods and analysis provided to allow replication by others?

Yes

Are all the source data and materials underlying the results available?

Yes

Reviewer Expertise:

Mining engineering

I confirm that I have read this submission and believe that I have an appropriate level of expertise to confirm that it is of an acceptable scientific standard, however I have significant reservations, as outlined above.

# Reviewer report: Reviewer response for version 1

**DOI:** 10.21956/openreseurope.21191.r63923

**Published:** 2025-12-10

**Authors:** Giovanna Antonella Dino

**Affiliations:** 1 University of Torino, Torino, Italy

The paper “ *Quarry mining or nature conservation: emerging conflicts in Serra de Aire e Candeeiros Natural Park (Portugal)*” is, overall, well written. The topic addressed is both relevant and timely, particularly in light of the social conflicts that may arise in areas—whether protected or not—affected by extractive activities. The manuscript offers valuable insights into these dynamics.

 Some specific comments are provided below.

**GENERAL COMMENTS:**

- I’d suggest to include a list of Acronyms used in the text
- General English revision

**INTRODUCTION:**

 I’d suggest to:

- use the terms “ornamental stones” or “dimension stones” instead of “ornamental rocks”
- cancel **€ **from €459,632. (10 ^3^ **^€^**)

**STUDY AREA**

 I’d suggest to:

- write “between 13°C and 15°C”, instead of “between 13°c and 15°c”

**METHODS**

 I’d suggest to:

- erase the **comma** in the sentence “2- *The second stage* ***,*** *considered the search for extractive companies operating in the region and data collection (semi-structured interviews)”.*

**RISULTS AND ANALYSIS**

 I’d suggest to:

- decide, in the sentence “ *Based on the interview questions, a list of 9 codes was generated (* *Table 1* *) with their own definitions, allowing the methodological variables to be analyzed to be specified*”, to use only “to be analyzed” (and erase “to be specified”).
- to update Figure 4. Word cloud, putting together area and areas; problem and problems; company and companies, recover and recovery

**DISCUSSION**

 I’d suggest to:

- summerize the period “ *In terms of market analysis, it was observed that companies export 90% of their production, leaving a limited margin for sales and distribution within the Portuguese national market. The Asian market is the primary purchaser of marble blocks extracted by the companies, with the residual material from the production process targeting the national market*.”

To change the sentence “ *In relation to the variable type of material, this demonstrates the manner in which mining is conducted in and around the park, employing an open pit mining technique with the use of explosive charges to create the initial excavation. This is followed by the implementation of diamond wire and water to exert pressure on the cut walls, resulting in the disintegration of the rock blocks and their subsequent transportation to the milling stage or direct sale in the market.” *Please make sure the description of the exploitation technique is accurate. The diamond wire machine likely creates benches and then cuts blocks that are processed in the working plants. Any portion that is not suitable — in terms of size — *may* become “waste rock,” but it can also be recovered as “armour stones” or as undersized blocks to be cut with a diamond framesaw

If applicable, is the statistical analysis and its interpretation appropriate?

Yes

Is the study design appropriate and is the work technically sound?

Yes

Is the work clearly and accurately presented and does it engage with the current literature?

Yes

Are the conclusions drawn adequately supported by the results?

Yes

Are sufficient details of methods and analysis provided to allow replication by others?

Yes

Are all the source data and materials underlying the results available?

Yes

Reviewer Expertise:

Mining and quarrying expert

I confirm that I have read this submission and believe that I have an appropriate level of expertise to confirm that it is of an acceptable scientific standard.

# Response

**Published:** 2025-12-23

**Authors:** María Guillermina Diaz

**Affiliations:** Mendoza, National Scientific and Technical Research Council, Mendoza ciudad, Mendoza, Argentina

We would like to express our sincere gratitude to Reviewer 2 for the positive evaluation of our manuscript and for the thoughtful and constructive suggestions, which have helped us improve both the clarity and technical accuracy of the paper. Below we detail how each comment was addressed:

 1.    General comments: **Response:**

- A List of Acronyms at the beginning of the paper for reader clarity would be a great help, we will check with the editors.
- A comprehensive English (UK) revision of the entire manuscript has been carried out to improve fluency, grammar, and terminology consistency.

2.    Introduction: **Response:**

- In accordance with the reviewer’s suggestion, we have replaced the term “ornamental rocks” with “ornamental stones” or “dimension stones” throughout the text.
- The symbol “€” was removed from “€459,632 (103€),” as recommended.

3.    Study area: **Response:**

- he temperature range now reads “between 13°C and 15°C,” correcting the lowercase “c” and improving consistency with scientific formatting standards.

4.    Methods: **Response:**

- The unnecessary comma was removed from the sentence “The second stage considered the search for extractive companies operating in the region and data collection (semi-structured interviews)” making the sentence more grammatically correct and fluid.

5.    Results and analysis: **Response:**

- We have simplified the sentence as advised, keeping only “to be analyzed” and deleting “to be specified.

6.    Discussion: **Response:**

- The market analysis paragraph has been summarized to focus on the key findings: the export orientation of production, the predominance of the Asian market, and the limited domestic distribution.
- The description of the exploitation technique has been revised to ensure technical accuracy. The new version clarifies that the quarrying process involves creating benches with diamond wire saws, cutting stone blocks that are subsequently processed in working plants. It also notes that portions of rock not suitable as blocks are treated as waste rock for abandoned quarry projects, as per the reviewer’s correction.

We are deeply grateful for the reviewer’s detailed technical insights, which have improved the scientific precision, structure, and readability of our manuscript.

# Response

**Published:** 2026-01-16

**Authors:** Luis SANTOS

Dear Dr. Giovanna Antonella Dino,  We thank you very much for the careful reading of the manuscript and for the constructive and valuable comments provided. All remarks and suggestions have been thoroughly considered and addressed in the revised version of the manuscript, which has been submitted to the journal’s editor. We believe that these revisions have improved the clarity and quality of the work. Best regards

# Reviewer report: Reviewer response for version 1

**DOI:** 10.21956/openreseurope.21191.r63922

**Published:** 2025-11-18

**Authors:** Mrutyunjay Padhiary

**Affiliations:** 1 Assam University, Silchar, India

This paper examines the socio-territorial conflicts between quarry mining and nature conservation and aims to address existing gaps in the literature regarding non-metalliferous mining and its socio-environmental impacts in this specific region.

- The introduction mentions a gap in non-metalliferous mining and socio-environmental implications in PNSAC; authors should specify how this study uniquely addresses it beyond merely examining conflicts.

- Please elaborate on the qualitative content analysis framework employed for semi-structured interviews, rather than merely indicating its application. Explain the criteria for identifying key stakeholders and how snowball sampling ensured a representative, rather than biased, selection.

- The results section is short; please add more details about the types of tensions and territorial disputes, such as specific examples or initial findings. Elaborate on the unequal distribution of environmental and social costs by providing examples or categories of these costs.

- Explain why mining doesn't create many local jobs despite economic activity, perhaps linking it to the advanced machinery mentioned elsewhere. The statement needs further elaboration or examples regarding how mining companies contribute to road improvements while the park facilitates mining expansion.
- Acknowledge the limitation of only five interviews more prominently in the discussion, especially given the complexity of the issue.
- The author may refer to 1 and cite it.

If applicable, is the statistical analysis and its interpretation appropriate?

Not applicable

Is the study design appropriate and is the work technically sound?

Yes

Is the work clearly and accurately presented and does it engage with the current literature?

Yes

Are the conclusions drawn adequately supported by the results?

Yes

Are sufficient details of methods and analysis provided to allow replication by others?

Yes

Are all the source data and materials underlying the results available?

Yes

Reviewer Expertise:

Precision agriculture, Environmental engineering, AI in farming, Food processing, Image processing in agriculture.

I confirm that I have read this submission and believe that I have an appropriate level of expertise to confirm that it is of an acceptable scientific standard, however I have significant reservations, as outlined above.

## References

[1] : Assessing the Environmental Impacts of Agriculture, Industrial Operations, and Mining on Agro-Ecosystems.1165: 10.1007/978-3-031-70102-3_8 107-126 10.1007/978-3-031-70102-3_8

# Response

**Published:** 2025-12-23

**Authors:** María Guillermina Diaz

**Affiliations:** Mendoza, National Scientific and Technical Research Council, Mendoza ciudad, Mendoza, Argentina

We sincerely thank the reviewer for their careful reading of our manuscript and for the constructive suggestions that have helped us significantly strengthen the paper. We have carefully addressed each point as detailed below:

 1.    Clarification of the study’s unique contribution

**Response: **We have revised the introduction to clearly state how our study uniquely addresses the identified research gap. Specifically, we emphasize that it goes beyond describing conflicts by analyzing the mechanisms through which socio-territorial tensions emerge in non-metalliferous quarrying areas within the PNSAC. The revised text highlights the novelty of integrating socio-environmental perspectives with regional territorial governance analysis.

 2.    Elaboration of the qualitative framework

**Response: **The methods section now includes a detailed explanation of the qualitative content analysis framework used for semi-structured interviews. We describe our coding process, criteria for category development, and how analytical themes were derived. Additionally, we explain the selection criteria for key stakeholders and clarify how the snowball sampling approach minimized bias by drawing from multiple institutional, local, and industry networks until thematic saturation was reached.

 3.    Expansion of the results section

**​​​​​​​Response: **The results section has been substantially expanded to include specific examples of tensions and disputes, such as conflicts over land use, regulatory interpretations, and compensation measures. We also provide more explicit categories of environmental and social costs (e.g., habitat fragmentation, landscape alteration, and perceived inequities in tourism-related benefits).

 4.    Economic and employment aspects

**​​​​​​​Response: **We now provide further explanation of the relationship between limited local job creation and increased mechanization in quarry operations, illustrating how technological advances reduce the need for manual labor. This section also explains the reciprocal interactions between quarry companies and local authorities regarding road maintenance and infrastructure improvements, detailing how these exchanges both mitigate and perpetuate mining activity.

 5.    Acknowledgement of limitations

**​​​​​​​Response: **We have made the limitation related to the small number of interviews (five) more explicit in the discussion section. The revised text acknowledges this constraint and discusses its implications for the scope of our conclusions while suggesting avenues for future research to expand stakeholder perspectives.

 6.    Reference to citation 1

**​​​​​​​Response: **​​​​​​​As suggested, we have referred to citation 1 where relevant and integrated it appropriately into the discussion. We believe these revisions comprehensively address the reviewer’s comments and have enhanced the analytical depth, methodological clarity, and contextual richness of the article. We thank the reviewer again for their valuable feedback, which has substantially improved the quality of our manuscript.

# Response

**Published:** 2026-01-16

**Authors:** Luis SANTOS

Dear Reviewer We sincerely thank the reviewer for the time and effort devoted to evaluating our manuscript and for the insightful and constructive feedback. All comments have been carefully addressed, and the manuscript has been revised accordingly. The updated version has been submitted to the journal’s editor, and we believe that your suggestions have significantly strengthened the manuscript. Regards
